# IMPROVE-DD: Integrating multiple phenotype resources optimizes variant evaluation in genetically determined developmental disorders

**DOI:** 10.1016/j.xhgg.2022.100162

**Published:** 2022-11-24

**Authors:** Stuart Aitken, Helen V. Firth, Caroline F. Wright, Matthew E. Hurles, David R. FitzPatrick, Colin A. Semple

**Affiliations:** 1 MRC Human Genetics Unit, Institute of Genetics and Cancer, University of Edinburgh, Edinburgh EH4 2XU, UK; 2 Wellcome Sanger Institute, Hinxton, Cambridgeshire CB10 1SA, UK; 3 Clinical Genetics Department, Addenbrooke’s Hospital Cambridge University Hospitals, Cambridge CB2 0QQ, UK; 4 University of Exeter Medical School, Royal Devon & Exeter Hospital, Barrack Road, Exeter EX2 5DW, UK

**Keywords:** human phenotype ontology, phenotype, genotype, developmental disease, growth, developmental milestones, genetic diagnosis

## Abstract

Diagnosing rare developmental disorders using genome-wide sequencing data commonly necessitates review of multiple plausible candidate variants, often using ontologies of categorical clinical terms. We show that Integrating Multiple Phenotype Resources Optimizes Variant Evaluation in Developmental Disorders (IMPROVE-DD) by incorporating additional classes of data commonly available to clinicians and recorded in health records. In doing so, we quantify the distinct contributions of sex, growth, and development in addition to Human Phenotype Ontology (HPO) terms and demonstrate added value from these readily available information sources. We use likelihood ratios for nominal and quantitative data and propose a classifier for HPO terms in this framework. This Bayesian framework results in more robust diagnoses. Using data systematically collected in the Deciphering Developmental Disorders study, we considered 77 genes with pathogenic/likely pathogenic variants in ≥10 individuals. All genes showed at least a satisfactory prediction by receiver operating characteristic when testing on training data (AUC ≥ 0.6), and HPO terms were the best predictor for the majority of genes, though a minority (13/77) of genes were better predicted by other phenotypic data types. Overall, classifiers based upon multiple integrated phenotypic data sources performed better than those based upon any individual source, and importantly, integrated models produced notably fewer false positives. Finally, we show that IMPROVE-DD models with good predictive performance on cross-validation can be constructed from relatively few individuals. This suggests new strategies for candidate gene prioritization and highlights the value of systematic clinical data collection to support diagnostic programs.

## Teaser

Diagnosing rare developmental disorders using genome-wide sequencing data requires review of multiple plausible candidate variants, often using ontologies of categorical clinical terms. We show that integrating multiple phenotype resources optimizes variant evaluation in developmental disorders (IMPROVE-DD) by incorporating additional phenotypic data commonly available to clinicians and recorded in health records.

## Main text

The importance of phenotype to ranking candidate disease-causing genes is established in research and increasingly so in clinical practice. The primary data resource used in computational phenotype analyses is the Human Phenotype Ontology (HPO).^1^ Despite promising results,^2^^,^^3^ the exploitation of other information readily available to clinicians, including quantitative anatomic measurements and patient images, is less prevalent.^4^ The HPO resource, HPO-encoded disease models, and patient’s disease descriptions in HPO terms support diverse tasks: protocols start with a systematic description of an individual’s phenotype and may progress to suggested diagnoses.^5^ While numerous computational and statistical approaches have been proposed, the advantages of *likelihood ratios* for the interpretation of genomic and phenomic data in rare disease have been demonstrated.^6^

Probabilistic methods for combining HPO terms with genetic data in Mendelian disease have been proposed,^7^^,^^8^^,^^9^ as have statistical criteria^10^^,^^11^^,^^12^ and deep learning,^13^^,^^14^ commonly as components of a variant prioritization workflow. Others have aimed to support users through ontology-assisted visualization and ranking.^15^^,^^16^^,^^17^^,^^18^ Here, we show that integrating multiple phenotype resource optimizes variant evaluation in developmental disorders (IMPROVE-DD) by utilizing a range of clinical datasets coupled with gold-standard diagnoses confirmed by clinical evaluation.

Probabilistic models are often compared through the likelihood ratio that uses Bayes rule to decompose the joint probability of the models under consideration ($M_{1}$ and $M_{2}$) and the data to the conditional probability of the data given the model and the prior:

(Equation 1) $$LR\left(M_{1},M_{2}\right)=\;P\left(D|M_{1}\right)P\left(M_{1}\right)/P\left(D|M_{2}\right)P\left(M_{2}\right)$$

This formulation avoids calculating the probability of observing the data *P(D)*, which can be difficult to evaluate.

The Deciphering Developmental Disorders (DDD) study recruited individuals with severe or extreme developmental disorders (DDs) in whom clinical assessment and baseline genetic investigation were unable to establish a molecular diagnosis.^19^^,^^20^^,^^21^^,^^22^ Whole exome sequencing (WES) was performed in >13,500 unrelated individuals with 85% analyzed as nuclear trios (affected child with both parents) and the remainder as singleton WES. Detailed phenotypic information (see below) was recorded by clinicians using the secure portal within the DECIPHER system^23^ (deciphergenomics.org). A combination of rational filtering for impactful genotypes at known DD loci and statistical genomic approaches to the discovery of novel loci and genetic mechanisms has proven to be successful in diagnosing in >33% of the cohort.

At the time of access, the DDD dataset included information on 13,439 individuals. In addition to age, sex, and HPO terms, the available clinical information included the following: five growth attributes, which were gestation (in months), birthweight, height, weight, and head circumference (expressed as Z scores); plus four developmental milestones (in months) marking when the child first walked independently, spoke their first words, expressed a social smile, and sat independently.

We used genetic diagnoses assigned by referring clinical centers as the ground truth and only considered individuals with a single diagnosis. Diagnoses in one of 856 genes were recorded for 4,112 individuals. However, we required at least 10 individuals per genetic disorder to build gene-disease models and a relatively complete record of quantitative data in order to be able to model a gene. This reduced the number of genes we could consider to 77 in 1,730 individuals. The median number of individuals per gene was 17 (maximum 81).

**Figure 1:**
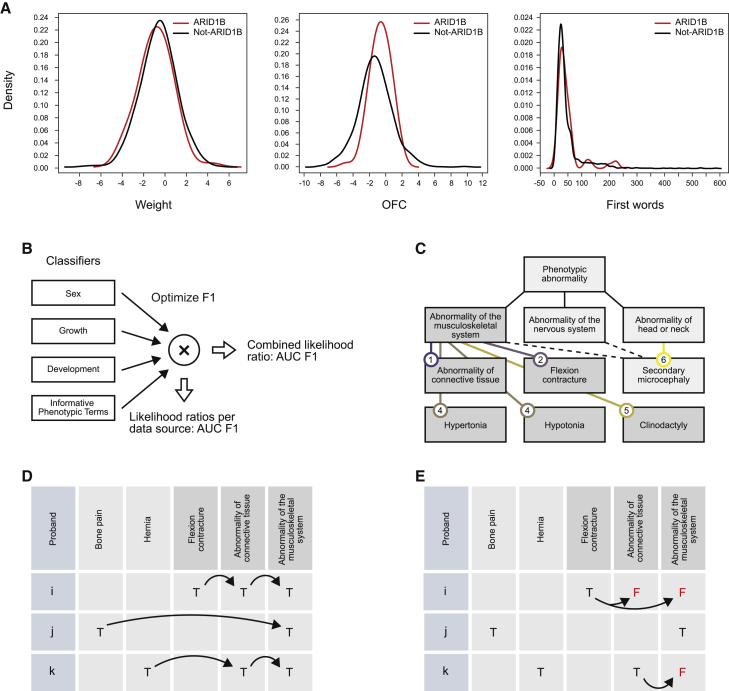
Integrating probabilistic models for nominal, continuous, and ontology annotation data (A) Continuous data for growth and development is modeled by a smoothed kernel density. (B) Classifiers for sex, growth, development, and HPO terms are constructed and evaluated individually, and they are combined by optimally weighting their output likelihoods. (C) All top-level HPO phenotypes (immediately below *Phenotypic abnormality*) are considered informative phenotypic terms (IPTs). Dependent on their annotation frequency across the cohort, child terms are selected as IPTs (250–1500 uses) or further expanded (>1,500 uses). IPTs such as *Secondary microcephaly* that can be selected by multiple paths are included under one top-level IPT only. The number of HPO levels a child term is below its top-level term is given at the end of the arc. (D) Annotations are propagated from child to parent terms (arrows) to expand the annotation matrix according to the HPO structure. (E) Annotations to IPTs that arise from propagation from an IPT are removed: individual *i* has *Flexion contracture* but not parents of this term, whereas individual *j* has *Abnormality of the musculoskeletal system* among the annotations to informative terms, as *Bone pain* is not selected as informative.

In IMPROVE-DD (web resources), we apply Bayesian methods to integrate diverse quantitative phenotypic data types and measure their contribution to decision-making. Here, decision-making is formalized as classifying each individual to the correct genetic diagnosis (for example, *ADNP* as the causal gene vs. not) to test whether the phenotype under consideration is both consistently observed and sufficiently distinct from that of the remaining DD cohort. We took advantage of the likelihood ratio approach to explore the contributions to making a diagnosis of each of the available data types available in DDD (sex, growth, development, and HPO annotations) by implementing a naive Bayes classifier for each gene from each data type (Figures 1A and 1B) using the R package naivebayes (web resources). Each classifier computes a likelihood ratio (Equation 1) comparing $M_{1}$ with $\bar{M_{1}}$, taking the prior as the observed frequency of each hypothesis in the data. A classifier for nominal data such as sex is simply a table of probabilities, while for continuous data, we used a smoothed kernel (the nrd0 kernel with increased bandwidth) to model the data. A feature will have diagnostic value when its distribution for $M_{1}$ differs from that of $\bar{M_{1}}$; for example, for *ARID1B*, head circumference (OFC) is discriminative, but weight is not (Figure 1A).

We next sought methods for selecting HPO terms and deriving useful probabilistic models from them in the likelihood framework. Information content (IC), defined as –log(probability of term),^24^ has been combined with genomic frequency and has been used to compare ontology-encoded phenotypes to aid prediction.^8^^,^^20^ When considering the entire corpus of annotations (to all 13,439 individuals whether diagnosed or not), the least frequently used terms are most informative but describe the fewest individuals. First, annotation frequencies for all terms, whether used directly in annotation or not, were found by propagating all annotations to their parent terms. A set of *informative phenotypic terms* (IPTs) was identified as follows: starting with the top-level terms for phenotypic abnormality that distinguish disorders of the major organ systems and developmental processes, each was expanded into a set of child terms that met minimal and maximal annotation frequency criteria across the entire cohort (2% and 10% respectively) (Figure 1C). Working in a top-down manner to preferentially select terms that balance IC with generality in the ontology graph, child terms with usage above the upper limit were expanded, and those within lower and upper limits were retained in the IPT set. As IPTs were identified by expanding terms under phenotypic abnormality, an IPT might be found by multiple paths. The IPT data structure was organized so each IPT occurred under a single top-level term, and where an IPT had a parent that was also an IPT, the annotation matrix was modified so annotation to the parent IPT was deleted for individuals with the child term (Figures 1D and 1E). This procedure and the scripts that implement it are described in more detail in the tutorial in File S1. The frequency of use of IPTs was found from the modified annotation matrix in a computationally efficient manner. Examples of informative phenotypic terms included Mild, Moderate, and Severe global developmental delay (HP:0011342, HP:0011343, HP:0011344) organized under top-level IPT Abnormality of the nervous system (HP:0000707) (Table 1, File S2).

**Table 1:** Examples of informative phenotypic terms

Abnormality of the nervous system (HP:0000707)	Abnormality of the cardiovascular system (HP:0001626)	Abnormality of limbs (HP:0040064)
Mild global developmental delay (HP:0011342)	Abnormal heart morphology (HP:0001627)	Abnormal 5^th^ finger morphology (HP:0004207)
Moderate global developmental delay (HP:0011343)	Abnormality of the vasculature (HP:0002597)	Abnormal thumb morphology (HP:0001172)
Severe global developmental delay (HP:0011344)	Abnormal cardiovascular system physiology (HP:0011025)	Abnormal fingertip morphology (HP:0001211)

This procedure uses term frequencies from annotations to all individuals irrespective of their diagnosis, seeking terms with moderate IC across the dataset. The resulting 157 terms were used as features in classifiers for all genes we modeled: their frequency of use was found from the modified annotation matrix for IPTs when it was factored by diagnosis (supplemental information).

**Figure 2:**
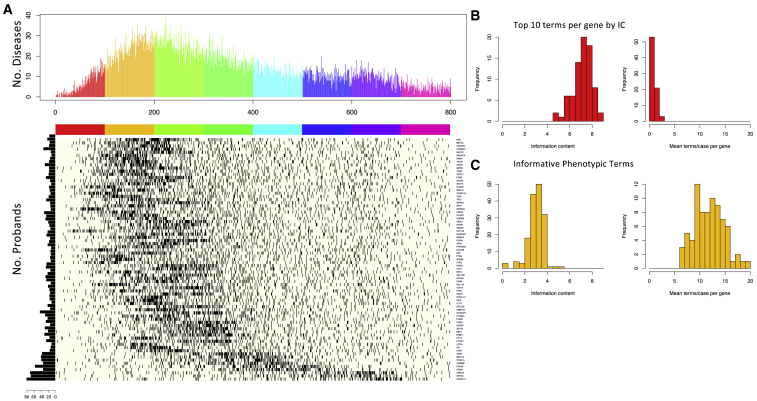
Informative phenotypic terms differ from the highest scoring terms by information theoretic criterion (A) Heatmap showing for each gene (row) the occurrence of an IPT in a ranking of HPO terms by TF IDF IC. HPO terms are ordered left to right by decreasing TF IDF IC. Top panel shows the number of diseases for which an informative term is found in rank *i* (from 1 to 800), and colors indicate scale, where each covers 100 positions. Although occurring toward the top of the 2,634 terms for which this measure can be calculated, IPTs are seldom in the top 50, and genes such as *ANKRD11*, *ARID1B*, and *KMT2A* in the bottom rows rank 400 and below. (B) Histograms of mean term IC (left) and mean number of individuals per term (right) when selecting the top 10 terms scored by TF IDF IC per gene. (C) Histograms of term IC (left) and number of individuals per term (right) for the 157 IPTs.

Measures from information retrieval including term frequency (TF) and inverse document frequency (IDF) have previously been adopted for the selection of relevant HPO terms.^25^ To compare our set of IPTs with those from an information theoretic (TF IDF IC) approach, we computed these measures for our dataset and examined the position of our IPTs in a ranking of terms per gene (Figure 2A). IPTs seldom ranked highest by TF IDF IC and can rank rather lowly, so they would not likely be selected by such a method. Terms ranking highly by TF IDF IC had considerably higher information content than IPTs, but considering the top 10 such terms, the number of terms per individual was low (Figure 2B). Applying the same ranking procedure to the terms that define an HPO gene model^1^^,^^26^(see web resources), we observed a more uniform ranking of HPO terms, where some terms ranked highly and others very lowly (Figure S1). We conclude that our approach uncovers a set of terms that are unlikely to have been selected by conventional metrics.

As summary statistics, we report the F1 score, being the harmonic mean of precision and recall, as a measure of the accuracy of decision-making, and area under the curve (AUC) as a measure of the rank of true positives irrespective of decision threshold across the entire dataset. The individual classifier outputs were combined by a set of weights, found per gene, that optimized F1 from the four input likelihoods plus an additional constant representing the prior. We discuss F1 and AUC from testing on the training data, as indicative of the potential of phenotype modeling, and results from a leave-one-out cross-validation that better controls for model overfitting.

**Figure 3:**
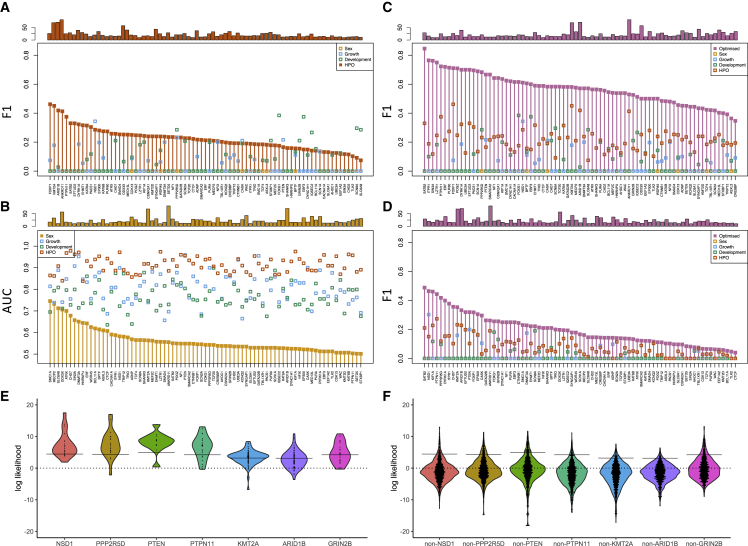
Integrating multiple phenotypic models improves classification (A) F1 per gene from sex, growth, development, and HPO classifiers (all values plotted at the same x coordinate). Vertical bars and filled symbols highlight the HPO classifier performance. Number of individuals per gene (upper). (B) AUC per gene highlighting sex. (C) F1 per gene highlighting the optimized score. (D) F1 per gene highlighting the optimized score when combining likelihoods from cross-validation. (E) Violin plots of likelihood ratios from HPO terms for selected genes (symbols are individuals). Likelihoods are shown for a prior of 0.5 to give a common zero reference line for all models. The per-gene prior is shown by the horizontal bar. The HPO model may represent a consensus for a majority of individuals, yet the model may not be sufficient to give a diagnosis for some individuals (*KMT2A*, *ARID1B*). Alternatively, the phenotypic spectrum may be broad (*NSD1*, *PPP2R5D*) and correctly diagnose all but a few cases. (F) Violin plots for the gene models in (E) applied to individuals whose diagnosis differs. A log likelihood above the per-gene prior is a false positive.

Beginning with the results from testing on the training data, the IPT-based HPO classifier had the best performance in decision-making for most genes (Figure 3A) with F1 scores of up to 0.46 when testing on training data. Exceptions were apparent; for example, growth was a better predictor for *NSD1*, while development was a better predictor for *GRIN2B* (Sotos syndrome MIM: 117550; GRIN2B MIM: 138252). Genes with larger numbers of individuals tended to score well when testing on training data: Pearson correlation between F1 from the HPO classifier and the number of individuals per gene was 0.61 (0.51 in cross-validation; p < 1e-5) indicating that performance was positively influenced by the number of individuals. In the interpretation of F1 scores, it should be noted that the prior probabilities $P\left(M_{i}\right)$ derived from the number of individuals range from 1/150 to 1/19, whereas the alternative hypotheses are much more probable (95/100 to 99/100). Consequently, the evidence from the data commonly failed to outweigh the prior, so precision or recall was 0, and F1 could not be calculated. We found the classification results were insensitive to the annotation frequency criteria used to select IPTs (supplemental information).

The best F1 scores from growth data alone were from *NSD1*, *PTEN*, and *DNMT3A* (Figures 3A, S2, and S3) with 20, 12, and 14 individuals, respectively (top three on cross-validation and top five when testing on training data). Turning to developmental milestones, the best F1 scores were from *SCN8A*, *FOXG1*, and *GRIN2B* (Figures 3A, S2, and S3) with 14, 21, and 25 individuals, respectively (top three by cross-validation and in the top eight testing on training data).

The best predicted genes from HPO annotations were *PTPN11*, *KMT2A*, and *ARID1B* with 25, 69, and 71 individuals, respectively (top three on leave-one-out cross-validation, top five when testing on training data). The likelihoods of individual IPTs can be examined for each individual and provide potentially useful diagnostic information to a clinician. We list the three most likely and three least likely *KMT2A* diagnoses according to phenotype modeling to show the balance of HPO terms for and against and, in the final case, the positive contribution from growth outweighed by the negative contribution from HPO terms (Table 2). Of note, the top three cases are pathogenic frameshifts, whereas the bottom three are likely pathogenic missense variants.

**Table 2:** Clinical evidence supporting or opposing the diagnosis of *KMT2A* in individuals with a pathogenic *de novo* variant

DECIPHER ID	Growth	Dev.	HPO	Total^a^	Informative phenotypic terms supporting *KMT2A*	Informative phenotypic terms opposing *KMT2A*
295774	2.5	2.0	18.2	12.9	Abnormal size of the palpebral fissures (1.4) Short stature (1) Intellectual disability mild (0.45) Abnormality of the endocrine system (0.44)	—
258419	5.0	0.8	16.2	12.2	Short stature (1) Abnormality of the endocrine system (0.44) Cognitive impairment (0.39)	—
294226	2.3	0.9	18.2	11.7	Abnormal size of the palpebral fissures (1.4) Short stature (1) Abnormal hair quantity (0.95) Abnormality of upper lip (0.41)	Abnormality of the nervous system (−0.011)
304702	−1.5	1.4	−1.3	−11.1	Epicanthus (0.88) Syndactyly (0.64) Abnormality of the genital system (0.23) Abnormality of prenatal development or birth (0.22)	Abnormality of the musculoskeletal system (−0.61) Abnormality of the forehead (−0.41) Abnormality of lower lip (−0.39) Abnormality of the fontanelles or cranial sutures (−0.37) Thick vermilion border (−0.37) Moderate global developmental delay (−0.34) Abnormality of toe (−0.017)
273901	−0.6	−0.4	−7.9	−19.3	Abnormal hair quantity (0.95) Abdominal symptom (0.45) Abnormality of eye movement (0.038)	Involuntary movements (−1.4) Generalized-onset seizure (−1.3) Abnormality of coordination (−0.82) Gait disturbance (−0.64) Dialeptic seizure (−0.46) Non-motor seizure (−0.28) Hypotonia (−0.22) Sleep disturbance (−0.2) Abnormality of the respiratory system (−0.041) Abnormality of the immune system (−0.02)
305957	5.7	−0.4	−17.6	−22.1	—	Abnormal ear physiology (−1.2) Abnormality of calvarial morphology (−1) Abnormality of skin pigmentation (−0.91) Abnormal emotion/affect behavior (−0.62) Abnormality of the musculoskeletal system (−0.61) Abnormality of the middle ear (−0.28) Localized skin lesion (−0.075)

a Values are log likelihood ratios. Note that the total includes values not listed here.

As would be expected, sex alone was a poor predictor, but this attribute has information by AUC for three genes: *SMC1A*, *MECP2*, and *DDX3X* (Figure 3B). A strong female bias is known for these X-linked syndromes (OMIM MIM: 300040; MIM: 300005; DDX3X MIM: 300160).

Performance measures from testing on the training data (the apparent error) are expected to be optimistic estimates of the true rates. In contrast, the estimates from leave-one-out cross-validation are believed to have less bias and more variance. To further explore issues of cross-validation, we performed a .632^27^ bootstrap cross-validation that combined the apparent error with that from held out samples in a bootstrap procedure. Due to the imbalance of the classes, the method could only be applied to the recall of the class of interest, which was shown to be intermediate between the apparent and leave-one-out values as would be expected (supplemental information).

We next optimized the F1 score for each gene by combining the likelihood ratios for each data type using five weights *w*_*0*_*–w*_*4*_ (Equation 2). For a given set of weights, a value from Equation 2 greater than 0 is a classification to the gene, from which true and false positives can be determined and F1 calculated.

(Equation 2) $${w_{0}\left(Ps\left(M_{1}\right)/Ps\left(\bar{M_{1}}\right)\right)}^{w_{1}}{\left(Pg\left(M_{1}\right)/Pg\left(\bar{M_{1}}\right)\right)}^{w_{2}}{\left(Pd\left(M_{1}\right)/Pd\left(\bar{M_{1}}\right)\right)}^{w_{3}}{\left(Ph\left(M_{1}\right)/Ph\left(\bar{M_{1}}\right)\right)}^{w_{4}}$$

In Equation 2, P(D|M) is abbreviated to Ps(M) for sex, and similarly for growth (*Pg*), development (*Pd*), and HPO (*Ph*). Simulated annealing implemented in the R package GenSA was used to find the optimal weights (web resources).

For all genes, optimization improved F1 over any individual data source (Figure 3C), achieving F1 scores greater than 0.7 for 12 genes when testing on training data. Genes with lower numbers of individuals were high in the ranking, indicating that good models can be found for them, but overfitting in the original model training may be at play. The F1 score was generally reduced in leave-one-out cross-validation where 14 genes had F1 0.3–0.5, namely, *ARID1B*, *CHD7*, *DYRK1A*, *EFTUD2*, *EP300*, *FOXP1*, *ITPR1*, *KIF1A*, *KMT2A*, *NSD1*, *PPP2R5D*, *PTEN, PTPN11*, and *SATB2* (Figures 3D and S3). Genes with larger numbers of individuals tended to have higher F1 (Pearson correlation 0.27; p = 0.015).

We also examined the distribution of likelihoods of a diagnosis (Figure 3E) versus all others (Figure 3F) from HPO gene models. This analysis highlighted a number of genes, including *KMT2A*, where the evidence from the fit to the HPO model did not outweigh the prior for many individuals and hence gave false negatives.

We then asked if an optimized HPO classifier would rival the combined data classifier. Optimal values were found for *w*_*0*_ and *w*_*4*_ (for the prior and HPO likelihood) using the same procedure. Using only HPO terms gave 147 fewer true positives (8.5% of the 1,730 individuals) and 2,041 more false positives in total (summing false positives over 77 genes). Per gene, median recall was reduced from 0.57 to 0.5, and median precision from 0.64 to 0.21. The benefits of additional phenotypic information, specifically growth and development data, are clear from these results. As an additional comparison, classifiers based on top terms by TF IDF IC and by disease model were also assessed and found to not perform as well as IPT-based classifiers (supplemental information).

**Figure 4:**
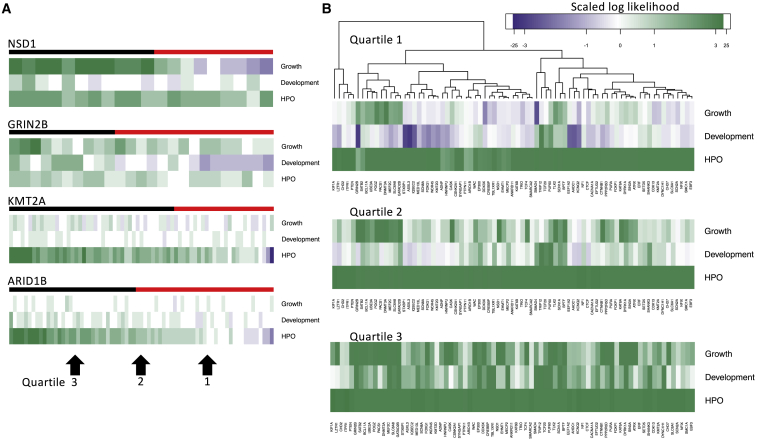
The contribution of data type to diagnosis varies by gene (A) Heatmaps of log likelihood per individual (column) for each data type (row) for selected genes. Values are scaled by optimization weight and columns ordered right to left from highest to lowest total likelihood (negative values in blue, positive in green). Upper bar shows true positives in black and false negatives in red. (B) Heatmaps of log likelihood multiplied by optimization weight per gene (column) for each data type (row). Heatmaps show values for individuals at quartiles 1, 2, and 3 successively for each data type. The hierarchical clustering reflects groupings at quartile 1. For example, for *GRIN2B*, the individual with the first quartile score fits the development model poorly (indicated in blue), and the median *GRIN2B* individual has a small negative contribution from development, and the contribution from development is positive by the third quartile. Only positive contributions are found from quartile 3, and HPO has a positive contribution at each quartile. The color scale truncates absolute values above 3 in order to focus on the range −3 to 3.

To further investigate the generality of each model in each data type, we assessed growth, development, and HPO models through their contribution to the optimized log likelihood for all individuals for each gene (Figure 4A). This revealed models that worked well in decision-making did not necessarily capture all diagnosed individuals; for example, growth in *NSD1* and development in *GRIN2B* captured distinctive subsets of individuals. To visualize this across all 77 genes, we selected individuals at quartiles 1, 2, and 3 representing poor, typical, and good fits to the gene models (Figure 4B). Where the scaled values were negative, the model contradicted the assigned diagnosis. We found HPO models agreed with the correct diagnosis at each quartile. Defining models to generalize well as those in which a positive likelihood ratio is found for the median individual, all but four growth models generalized well. However, development models for 22 genes (29%) failed to generalize by this criterion.

In summary, we found that HPO terms are the best predictor of a correct diagnosis for most genes, but 17% (13/77 genes) were better predicted by growth or development, and prediction from combined data performed better than any individual source. Predictions from combined data also gave notably fewer false positives than prediction from HPO terms alone, as median precision increased to 0.64 from 0.21 when only HPO terms were used. While more individuals are to be preferred when building models, we found gene models with good predictive performance on cross-validation could be built from a relatively small number of individuals (n ≥ 10). The derivation of likelihood ratios from individual annotations using HPO-encoded disease models of LIRICAL^6^ is closest to our approach. However, rather than define HPO models per gene, we began with the extensive database of individual annotations of the DDD study from which we are able to assess HPO term usage in clinical practice irrespective of diagnosis: our disease models are then computed from the observed annotations for each gene.

Bayesian methods are recognized as adding quantitative rigor to the combination of evidence for and against variant pathogenicity in rare disease while making explicit any assumptions regarding strength of evidence and disease prevalence.^28^ In addition, they are responsive to changes in the evidence base as new observations are made. We extend the application of this paradigm to phenotypic data making use of the extensive acquisition of growth measures and developmental milestones in addition to HPO terms in the DDD study. This approach could be extended by the incorporation of additional phenotypic data (e.g., images, epigenomic profiles, biochemical assays, etc.) to further improve gene-disease models and make them more applicable to other rare disorders. Although phenotypic models are unlikely to be sufficiently predictive by themselves, particularly for genetically heterogeneous disorders such as DD, they can be used to update posterior probabilities found from genomic analyses of variant pathogenicity^28^ and thus have an important role in increasing the robustness of a diagnosis. This is illustrated for an individual with a missense variant in *NSD1* with weak genetic evidence that could be strengthened to likely pathogenic through the likelihood ratio from HPO terms by the methods reported here.^29^

Quantitative patient data is of importance to clinical interpretation, and we have proved its value in computational disease modeling; however, systematic collection of and computational access to such data is often lacking in health data systems, potentially hindering diagnostic and biological insights.

## Data Availability

The code used to identify informative HPO terms and to run the classification and optimization procedures can be found in the IMPROVE-DD github repository. Sequence and variant-level data and phenotypic data for the DDD study data are available from the European Genome-phenome Archive (EGA; https://www.ebi.ac.uk/ega/) with study ID EGAS00001000775. Clinically interpreted variants and associated phenotypes from the DDD study are available through DECIPHER (https://www.deciphergenomics.org/).

## Web resources

DECIPHER, https://www.deciphergenomics.org/

EGA, https://www.ebi.ac.uk/ega/

genSA, https://CRAN.R-project.org/package=GenSA.

HPO https://hpo.jax.org/app/

HPO, Gene Models http://purl.obolibrary.org/obo/hp/hpoa/genes_to_phenotype.txt.

IMPROVE-DD scripts, https://github.com/Stuart-Aitken/IMPROVE-DD.

Naivebayes, https://CRAN.R-project.org/package=naivebayes

## Supplemental information

File S1. Tutorial, Supplemental methods, and Figures S1–S7

File S2. Excel file of informative phenotypic terms

Document S2. Article plus supplemental information
