# Supplementary material for: IMPROVE-DD: Integrating multiple phenotype resources optimizes variant evaluation in genetically determined developmental disorders
Source: HGG Adv. 2022 Nov 24;4(1):100162. doi: 10.1016/j.xhgg.2022.100162 (PMC9763511; doi:10.1016/j.xhgg.2022.100162)
Supplement: File S1. Tutorial, Supplemental methods, and Figures S1–S7 [file mmc1.pdf]

HGGA, Volume *4*

Supplemental information

IMPROVE-DD: Integrating multiple phenotype resources optimizes variant evaluation in genetically determined developmental disorders

Stuart Aitken, Helen V. Firth, Caroline F. Wright, Matthew E. Hurles, David R. FitzPatrick, and Colin A. Semple

Tutorial on creating sets of Informative Phenotypic Terms

Finding IPTs in the DDD database

Informative Phenotypic Terms (IPTs) were derived from a binary matrix of terms annotated to all DDD individuals (individuals in rows, HPO terms in columns) and an OBO format version of the HPO ontology (that of 02/08/2021). An extended matrix of propagated annotations was then created (direct annotations were propagated to parent terms using the ontology structure). The usage of HPO terms was then found for all HPO terms making no distinction between direct and inferred use.
Beginning with top level phenotypic terms (those under "Phenotypic abnormality" HP:0000118), all such terms were included as IPTs, and each was descended in turn to select subterms meeting the criterion of use in the extended annotation matrix (above 250 and below 1500 uses in the entire DDD dataset of 13439 individuals). Subterms with usage above the upper threshold were further expanded, and the criterion applied at that level. As there can be multiple paths to a term from the top level, an IPT can be found under multiple top level terms – these were detected and the IPT retained under a single top level term (in a list of lists data structure). The final step was to detect IPTs that, through the ontology structure, had parent terms that were also IPTs. To remove the resulting correlation in annotation of the parent to the child, the extended annotation matrix was modified to remove the annotation to the parent term for individuals with an annotation to the child IPT in question. This modified matrix was used to compute term frequencies in gene models.
The procedure does not depend on any specific version of HPO. The version of HPO specified can be found here: https://bioportal.bioontology.org/ontologies/HP

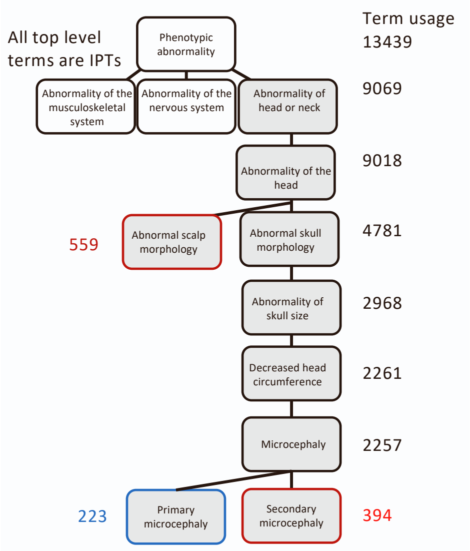

HPO terms were expanded (black) or retained as IPTs (red) according to usage.

Running the resource creation scripts

The following steps are run once for a database of HPO annotations made to individuals, generating a matrix that is used by the HPO classifier. This procedure does not require individuals to have diagnoses (diagnoses are considered in model learning and classification).
Implementing the steps described above, the IMPROVE_resource.r code begins by loading an OBO format of the HPO ontology using the ontoCAT R library (line 27).
In place of the DDD phenotype data (which is available on request), a matrix of individual annotations *hpo* is created from the data in *pheno* (line 67) after finding all terms used in annotation *allHPO*. The following steps can be run on data from another database providing the individual to HPO annotation is expressed as a binary matrix (individuals in rows, HPO terms in columns).
The expanded matrix *hpoe* is created from *hpo* using the *expandToParents()* method of the ontoCAT library (line 101). The entire set of expanded terms (*allHPOExpanded*) is found in a preliminary step in order to define *hpoe*.
The data structure *informativeSpecificTerms* is initialised from terms under HP:0000118 ("Phenotypic abnormality") and each top level term descended to identify terms meeting usage < *upper_annotation_threshold* and >= *retain_threshold* (lines 126 onwards). In the DDD data, we found 10% and 2% of total annotations were suitable thresholds.
As there can be multiple paths to an IPT, multiple instances may occur in *informativeSpecificTerms*. These are detected by tabluating terms and removing second and third occurrences to create *informativePhenotypicTerms*. This was performed intially after manual inspection (line 196) but is now automated (line 154).
The method *getDuplicatesAndParents()* returns information on any child-parent edges within *informativePhenotypicTerms* (line 255). A modified annotation matrix *hpou* is created from *hpoe* by removing parent annotation in individuals where there is an annotation to the child (lines 270). The *hpou* matrix is the resource used in the classifier. The HPO itself is not needed in classification

Methods

Classification and optimisation

Classifiers for nominal and continuous data were implemented using the naivebayes R package (Web Resources). For all growth and development data, the bandwidth of the nrd0 kernel to was increased to 1.5 to remove overfitting.
Optimisation was performed using the genSA R package (Web Resources). The inputs to optimisation were the likelihood ratios calculated by the models for each data source for all individuals. As the prior had been accounted for in all four cases, the priors were balanced to equality between the gene model and the alternative (as in **Figure 3E** and **3F**) and w_0 included in the optimisation as replacement. Optimisation maximised F1 by combining the likelihood values through the five weights as defined in equation (2).
A sensitivity analysis of parameter values around the optimal (replacing all parameters in each of 1000 iterations by sample from a normal distribution mean equal to the optimal, sd = 1/10 |optimal|) resulted in F1 values narrowly distributed around a reduced mean (**Figure S5**). The relatively small reduction in F1 indicates that non-optimal parameters cause the mis-classification of only a small number of cases.

Term selection

Annotations to HPO terms directly assigned to probands (4182 terms were used across 13439 individuals with a median of 6 terms per proband) were propagated to all parent terms, expanding the annotation to 5153 terms with a median of 40 terms per proband.
157 informative phenotypic terms were selected according to usage as decribed in the main text (**Figure 1D** and **1E**) with a median use of 10 terms per proband. Two alternative thresholds for term inclusion were explored: i. above 500 and below 3000 annotations resulting in 79 terms; and ii. above 125 and below 750 annotations resulting in 268 terms. Recall that the thresholds we adopted were above 250 and below 1500 annotations which gave 157 terms. The alternatives double and halve these thresholds respectively. The thresholds we selected give significantly better AUCs than the smaller set of 79 terms (p= 5.6e-06) although the average effect is small (0.03). There is no statistical difference between AUCs from 268 terms and 157 terms. Scatter plots of these results are now presented (**Figure S6)**. Consequently, we concluded that the choice of thresholds was not critical.

Term probabilities

Gene models were defined by the probabilities of the 157 IPTs in the annotations to diagnosed individuals *D*. The probability of each *IPT* in *D* was given by the m-estimate:

$$P(IPT) = {A(IPT,D)+1}\Big/{\left(\sum_{t=1}^{t=157} A(t,D)\right)+100}$$

where the term usage $A(t,D)$ was calculated from the modified annotation matrix as the sum over *t* (column in matrix) for probands (rows) assigned to *D*. The denominator normalises the counts to a specific term by the total annotations made to the 157 IPTs plus a number representing the vocabulary size.
When computing the probability of a set of IPT annotations in a case C_i for a given gene model, the product of probabilities was scaled to adjust for the number of IPTs used in this case. Where C_i had m IPTs the geometric mean probability was raised to the power of 10 (the median number of IPTs per case):

$$P(C_i\ GeneModel) = \prod_{j=1}^{m} P(IPTj)^{10/m}$$

This scaling made the resulting probabilities comparable across probands.
To investigate the impact of alternative term selection strategies, classifiers based on terms selected by TF IDF IC, or from HPO disease models were evaluated. In both cases, the 50 most informative terms per gene were selected (disease model terms were ordered by TF IDF IC) and term probability was based on term frequency in disease with Laplace smoothing:

$$P(HPO) = {A(HPO,D)+1}\Big/{(|D|+2)}$$

AUC and F1 were typically lower in gene-specific classifiers than for IPT classifiers when testing on training data (**Figure S4**). We found the occurrence of an HPO term of the gene model in a case to essentially guarantee classification to that model: Mean recall was high (0.79) showing that DDD cases indeed matched these disease models but mean precision was low (0.03). Even though we

selected terms with the highest IC per gene, they occurred in other diagnoses giving many false positives.

.632 bootstrap cross-validation

Performance measures are expected to be optimistic estimates of the true rates when testing on the training data, in contrast, the estimates from leave-one-out cross-validation are believed to have less bias and more variance. The error rate from testing on the training data, known as the apparent error, can be combined with the error from bootstrap resampling (where the error will be a pessimistic estimate to a known degree) to get a more balanced estimate using the .632 method due to Efron (1983). We derived bootstrap estimates for recall based on 17200 samples (ten times the dataset size) for samples absent from the bootstrapped data (sampling with replacement). As there was a large imbalance between the classes in each classification task we used the approach to estimate *recall* of the true positives for gene only, rather than include the much larger number of true negatives in the measure *accuracy*. The results of the .632 cross validation (**Figure S6**) show for HPO data alone, .632 values of recall per gene are mostly intermediate between the apparent error and leave-one-out estimates as would be expected. For the growth and development classification tasks there are many cases where recall is zero (as was the case for F1 by both cross-validation approaches) and again the .632 estimates of recall are mostly intermediate between the other estimates. This analysis indicates leave-one-out may be unduly pessimistic.

Efron, B. (1983) Estimating the Error Rate of a Prediction Rule: Improvement on Cross-Validation *Journal of the American Statistical Association* June 1983, Volume 78, Number 382.

A

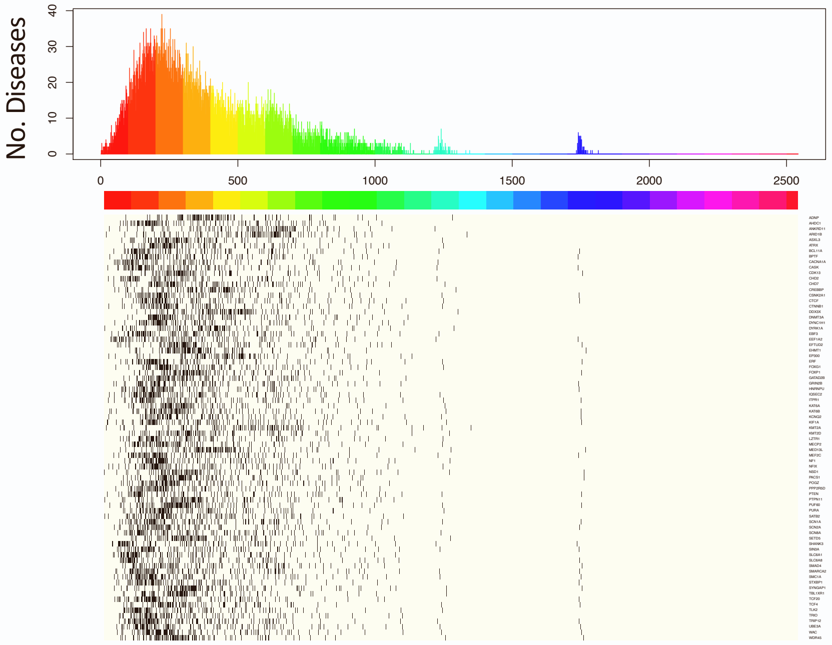

B

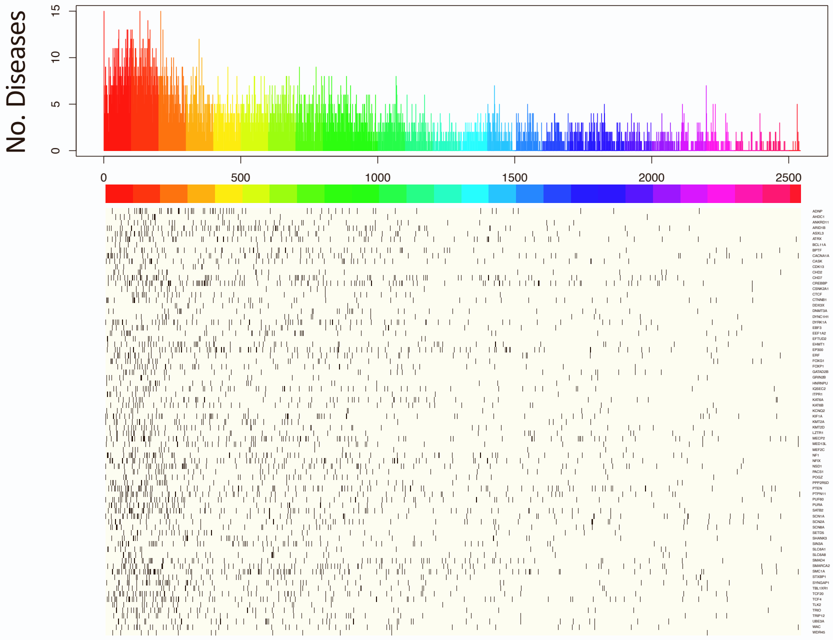

Figure S1. Disease model term ranking

A) Heatmap showing for each gene (row) the occurrence of an informative phenotypic term (IPT) in a ranking of all HPO terms by TF IDF IC. HPO terms are ordered left to right by decreasing TF IDF IC. Top panel shows the number of diseases for which a disease model term is found in rank i (from 1 to 2500), colours indicate scale, each covers 100 positions. Row order is alphabetical by gene name.

B) Heatmap showing for each gene (row) the occurrence of an term in the disease model for that gene in a ranking of all HPO terms by TF IDF IC.

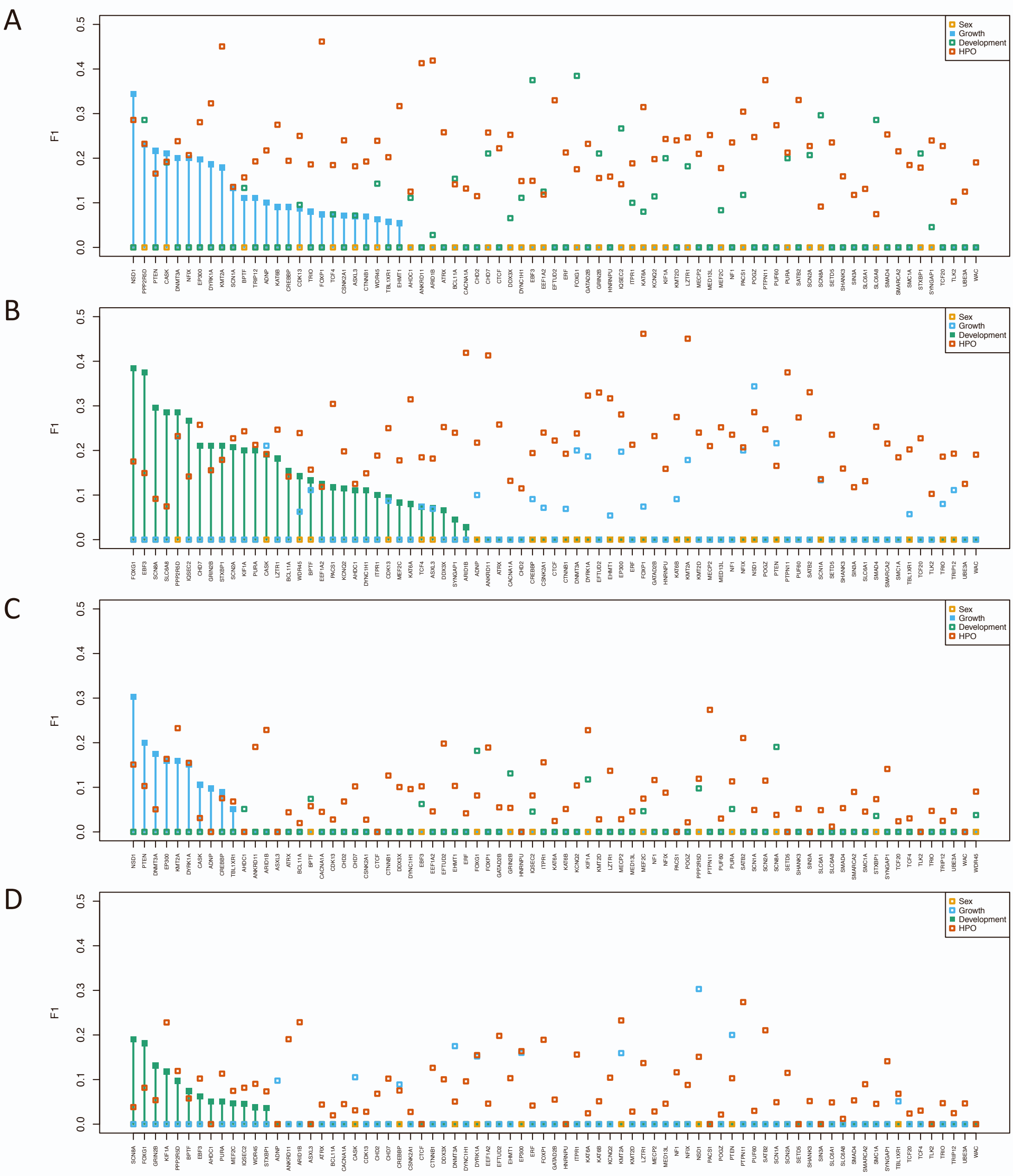

Figure S2. Classifier performance.

A) F1 per gene, testing on training data, ordered by the performance in growth (highlighted by the vertical bars and filled symbols) and B) ordered by performance in development.

C) F1 per gene, from a leave-one-out cross-validation, ordered by the performance in growth (highlighted by the vertical bars and filled symbols), and D) ordered by performance in development.

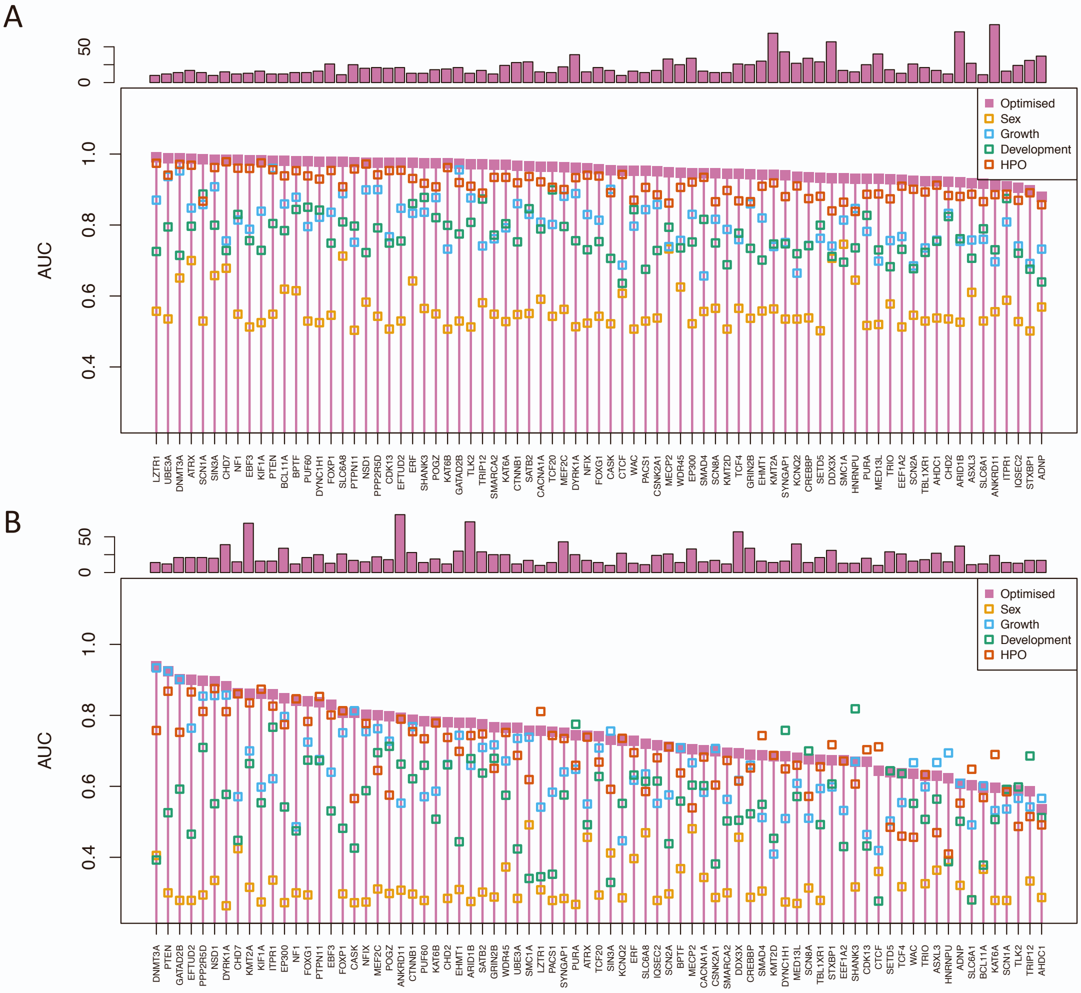

Figure S3. AUC on optimisation.
A) AUC from all data types when testing on training data is shown by symbols, filled symbols and vertical lines highlight the AUC after optimising for F1.
B) AUC from all data types in cross-validation is shown by symbols, filled symbols and vertical lines highlight the AUC after optimising for F1.

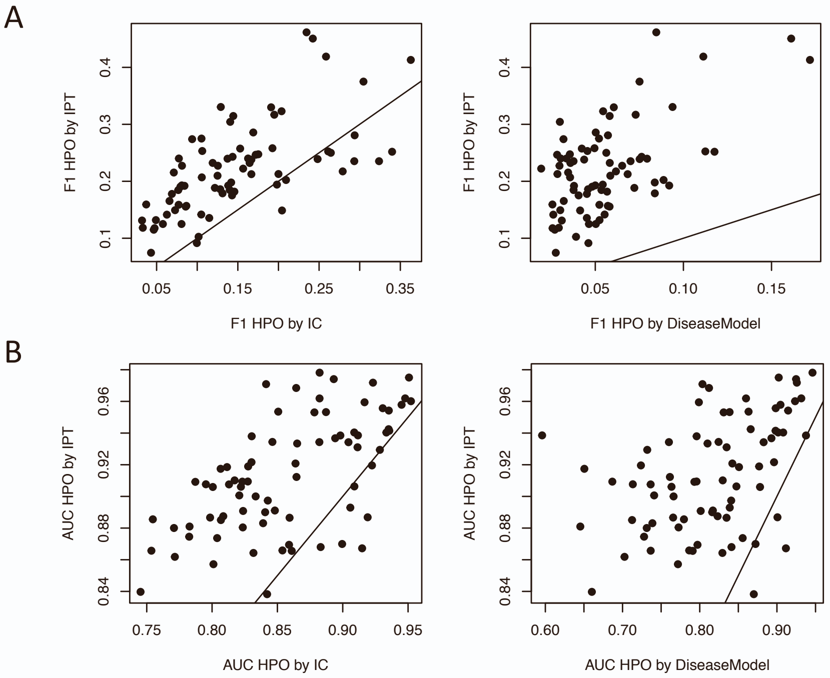

Figure S4. Comparison between IPT and alternative term sets for classification.

A) Scatterplot of F1 from IPTs against F1 from the top 50 terms per gene selected by TF IDF IC (left), and against the top 50 disease model terms per gene selected by TF IDF IC (right). Symbols are genes, lines show y=x.

B) Scatterplot of AUC from IPTs against AUC from the top 50 terms per gene selected by TF IDF IC (left) and against the top 50 disease model terms per gene selected by TF IDF IC (right). Symbols are genes, lines show y=x.

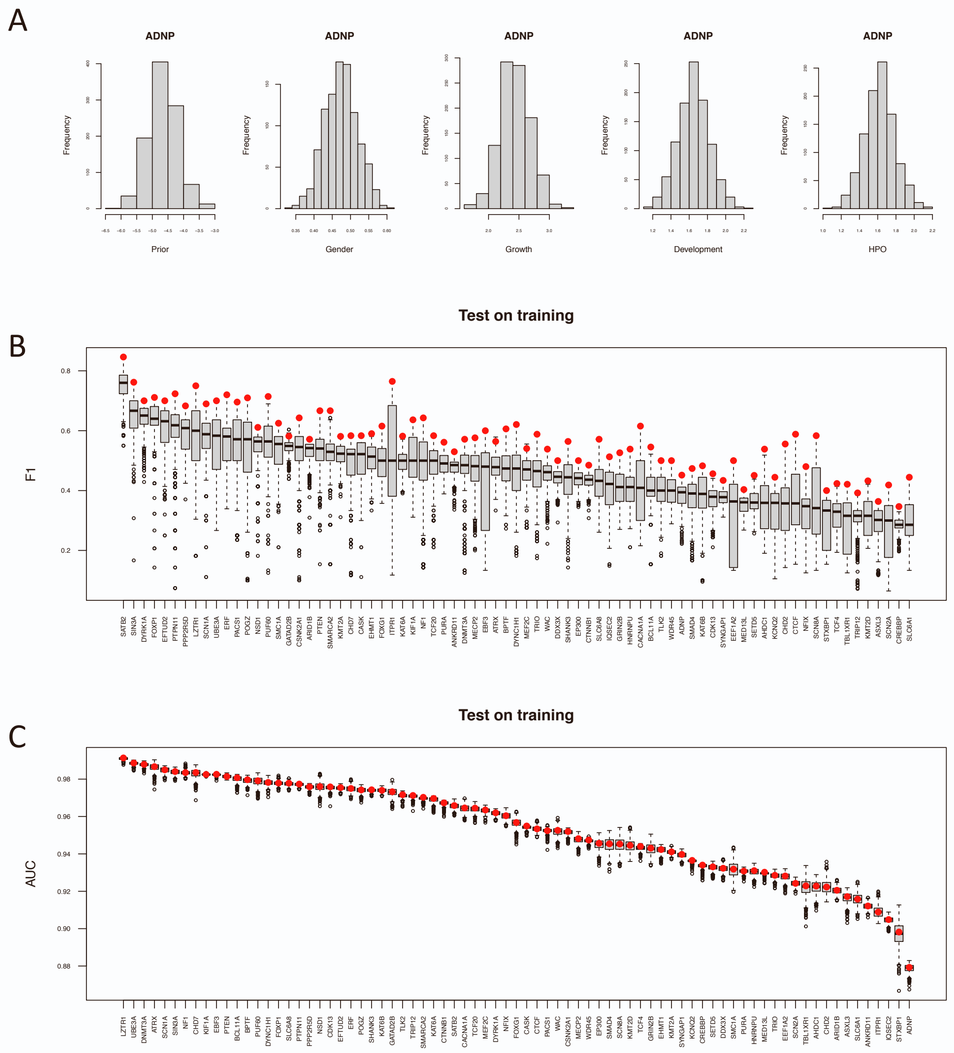

Figure S5. Sensitivity analysis.
A) Histograms of parameter values used in the sensitivity analysis of ADNP.
B) Boxplot of F1 per gene from 1000 random samples of the optimisation weights. F1 from optimal weights in red.
C) Boxplot of AUC per gene from 1000 random samples of the optimisation weights. AUC from optimal weights in red.

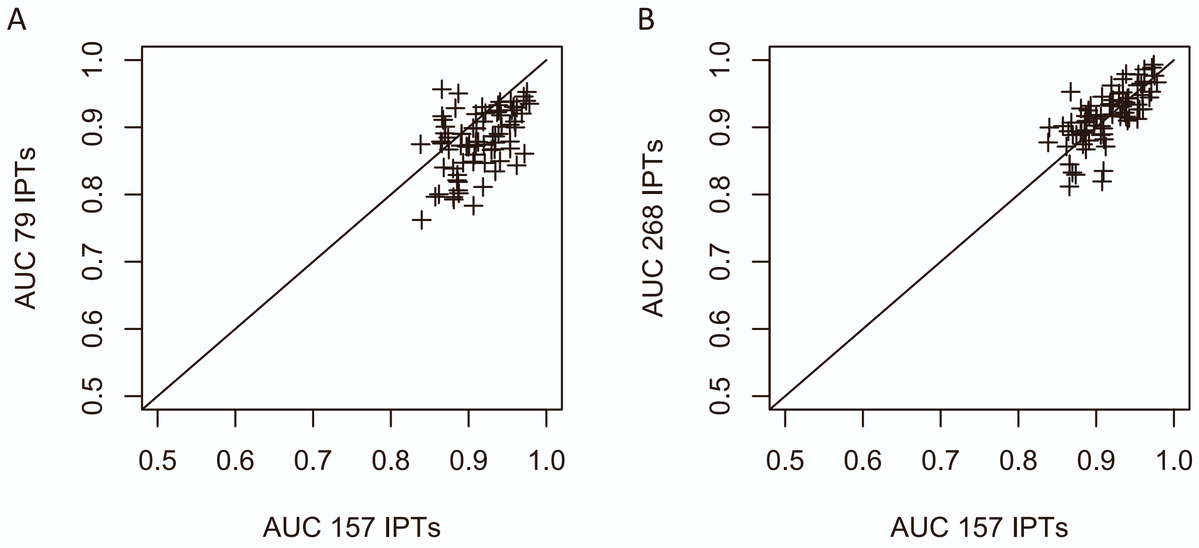

Figure S6. Comparison of alternative thresholds for IPT selection shows AUC per gene is insensitive to parameter choice

A) AUC computed from 79 IPTs selected from HPO terms with usage 500 to 3000 compared with 157 IPTs with usage 250 to 1500. Symbols are genes.

B) AUC computed from 268 IPTs selected from HPO terms with usage 125 to 750 compared with 157 IPTs with usage 250 to 1500. Symbols are genes.

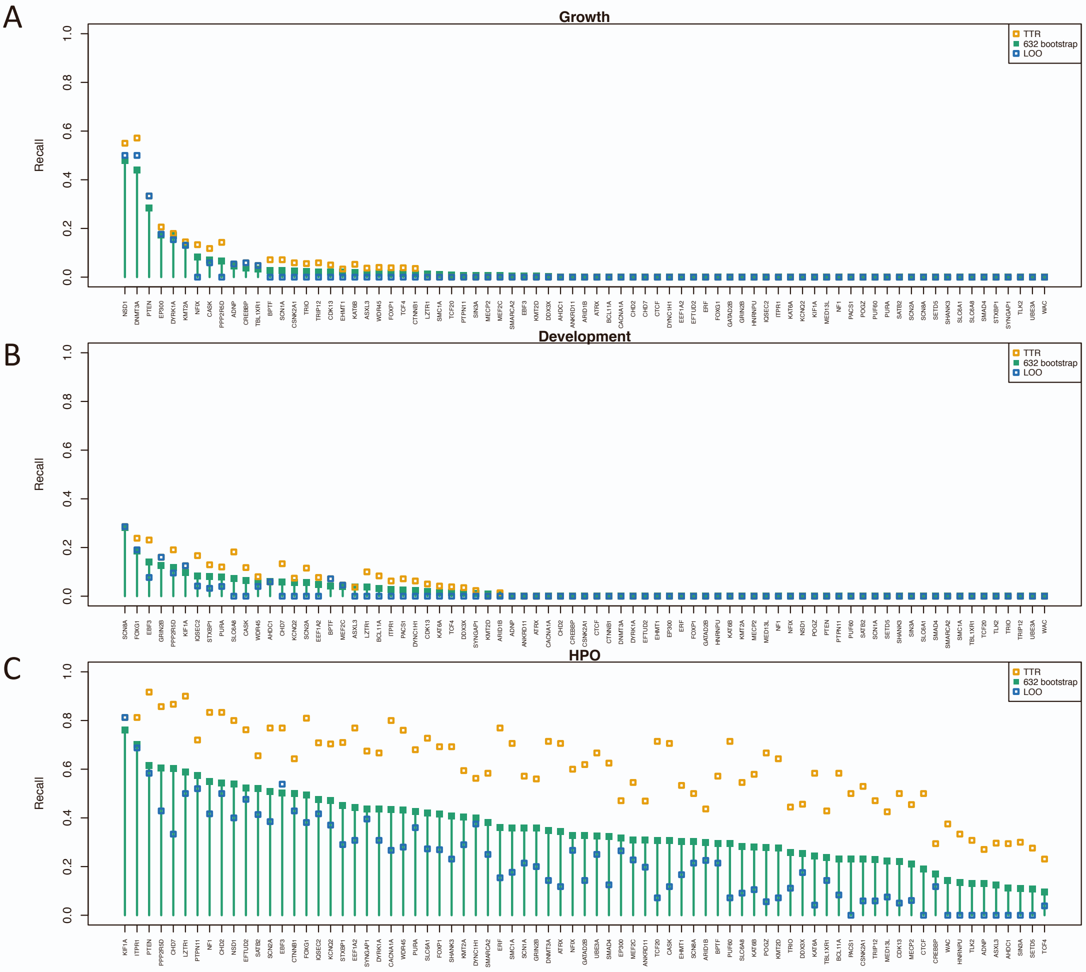

Figure S7. Bootstrap cross-validation shows a balanced estimate of recall lies between testing on training and leave-one-out rates.

A) Recall per gene using growth data alone when testing on training data, leave-one-out and by .632 bootstrap. Vertical bars and filled symbols highlight .632 recall.

B) Recall per gene using development data alone when testing on training data, leave-one-out and by .632 bootstrap. Vertical bars and filled symbols highlight .632 recall.

C) Recall per gene using HPO data alone when testing on training data, leave-one-out and by .632 bootstrap. Vertical bars and filled symbols highlight .632 recall.
