# Supplementary material for: IMPROVE-DD: Integrating multiple phenotype resources optimizes variant evaluation in genetically determined developmental disorders
Source: HGG Adv. 2022 Nov 24;4(1):100162. doi: 10.1016/j.xhgg.2022.100162 (PMC9763511; doi:10.1016/j.xhgg.2022.100162)
Supplement: Document S2. Article plus supplemental information [file mmc3.pdf]

HGG Advances

REPORT

IMPROVE-DD: Integrating multiple phenotype resources optimizes variant evaluation in genetically determined developmental disorders

Stuart Aitken,[1,6] Helen V. Firth,[2,3] Caroline F. Wright,[4] Matthew E. Hurles,[2] David R. FitzPatrick,[1,5] and Colin A. Semple[1,5,*]

Summary

Diagnosing rare developmental disorders using genome-wide sequencing data commonly necessitates review of multiple plausible candidate variants, often using ontologies of categorical clinical terms. We show that Integrating Multiple Phenotype Resources Optimizes Variant Evaluation in Developmental Disorders (IMPROVE-DD) by incorporating additional classes of data commonly available to clinicians and recorded in health records. In doing so, we quantify the distinct contributions of sex, growth, and development in addition to Human Phenotype Ontology (HPO) terms and demonstrate added value from these readily available information sources. We use likelihood ratios for nominal and quantitative data and propose a classifier for HPO terms in this framework. This Bayesian framework results in more robust diagnoses. Using data systematically collected in the Deciphering Developmental Disorders study, we considered 77 genes with pathogenic/likely pathogenic variants in $\geq$10 individuals. All genes showed at least a satisfactory prediction by receiver operating characteristic when testing on training data (AUC $\geq$ 0.6), and HPO terms were the best predictor for the majority of genes, though a minority (13/77) of genes were better predicted by other phenotypic data types. Overall, classifiers based upon multiple integrated phenotypic data sources performed better than those based upon any individual source, and importantly, integrated models produced notably fewer false positives. Finally, we show that IMPROVE-DD models with good predictive performance on cross-validation can be constructed from relatively few individuals. This suggests new strategies for candidate gene prioritization and highlights the value of systematic clinical data collection to support diagnostic programs.

The importance of phenotype to ranking candidate disease-causing genes is established in research and increasingly so in clinical practice. The primary data resource used in computational phenotype analyses is the Human Phenotype Ontology (HPO).[1] Despite promising results,[2,3] the exploitation of other information readily available to clinicians, including quantitative anatomic measurements and patient images, is less prevalent.[4] The HPO resource, HPO-encoded disease models, and patient's disease descriptions in HPO terms support diverse tasks: protocols start with a systematic description of an individual's phenotype and may progress to suggested diagnoses.[5] While numerous computational and statistical approaches have been proposed, the advantages of *likelihood ratios* for the interpretation of genomic and phenomic data in rare disease have been demonstrated.[6]

Probabilistic methods for combining HPO terms with genetic data in Mendelian disease have been proposed,[7–9] as have statistical criteria[10–12] and deep learning,[13,14] commonly as components of a variant prioritization workflow. Others have aimed to support users through ontology-assisted visualization and ranking.[15–18] Here, we show that integrating multiple phenotype resource optimizes variant evaluation in developmental disorders (IMPROVE-DD) by utilizing a range of clinical datasets coupled with gold-standard diagnoses confirmed by clinical evaluation.

Probabilistic models are often compared through the likelihood ratio that uses Bayes rule to decompose the joint probability of the models under consideration (M_1 and M_2) and the data to the conditional probability of the data given the model and the prior:

$$LR(M_1, M_2) = P(D|M_1)P(M_1)/P(D|M_2)P(M_2) \quad \text{(Equation 1)}$$

This formulation avoids calculating the probability of observing the data *P(D)*, which can be difficult to evaluate.

The Deciphering Developmental Disorders (DDD) study recruited individuals with severe or extreme developmental disorders (DDs) in whom clinical assessment and baseline genetic investigation were unable to establish a molecular diagnosis.[19–22] Whole exome sequencing (WES) was performed in >13,500 unrelated individuals with 85% analyzed as nuclear trios (affected child with both parents) and the remainder as singleton WES. Detailed phenotypic information (see below) was recorded by clinicians using the secure portal within the DECIPHER system[23] (deciphergenomics.org). A combination of rational filtering

[1]MRC Human Genetics Unit, Institute of Genetics and Cancer, University of Edinburgh, Edinburgh EH4 2XU, UK; [2]Wellcome Sanger Institute, Hinxton, Cambridgeshire CB10 1SA, UK; [3]Clinical Genetics Department, Addenbrooke's Hospital Cambridge University Hospitals, Cambridge CB2 0QQ, UK; [4]University of Exeter Medical School, Royal Devon & Exeter Hospital, Barrack Road, Exeter EX2 5DW, UK
[5]These authors contributed equally
[6]Lead contact
*Correspondence: colin.semple@ed.ac.uk
https://doi.org/10.1016/j.xhgg.2022.100162.
© 2022 The Authors. This is an open access article under the CC BY license (http://creativecommons.org/licenses/by/4.0/).

for impactful genotypes at known DD loci and statistical genomic approaches to the discovery of novel loci and genetic mechanisms has proven to be successful in diagnosing in >33% of the cohort.

At the time of access, the DDD dataset included information on 13,439 individuals. In addition to age, sex, and HPO terms, the available clinical information included the following: five growth attributes, which were gestation (in months), birthweight, height, weight, and head circumference (expressed as Z scores); plus four developmental milestones (in months) marking when the child first walked independently, spoke their first words, expressed a social smile, and sat independently.

We used genetic diagnoses assigned by referring clinical centers as the ground truth and only considered individuals with a single diagnosis. Diagnoses in one of 856 genes were recorded for 4,112 individuals. However, we required at least 10 individuals per genetic disorder to build gene-disease models and a relatively complete record of quantitative data in order to be able to model a gene. This reduced the number of genes we could consider to 77 in 1,730 individuals. The median number of individuals per gene was 17 (maximum 81).

In IMPROVE-DD (web resources), we apply Bayesian methods to integrate diverse quantitative phenotypic data types and measure their contribution to decision-making. Here, decision-making is formalized as classifying each individual to the correct genetic diagnosis (for example, *ADNP* as the causal gene vs. not) to test whether the phenotype under consideration is both consistently observed and sufficiently distinct from that of the remaining DD cohort. We took advantage of the likelihood ratio approach to explore the contributions to making a diagnosis of each of the available data types available in DDD (sex, growth, development, and HPO annotations) by implementing a naive Bayes classifier for each gene from each data type (Figures 1A and 1B) using the R package naivebayes (web resources). Each classifier computes a likelihood ratio (Equation 1) comparing M_1 with $\overline{M_1}$, taking the prior as the observed frequency of each hypothesis in the data. A classifier for nominal data such as sex is simply a table of probabilities, while for continuous data, we used a smoothed kernel (the nrd0 kernel with increased bandwidth) to model the data. A feature will have diagnostic value when its distribution for M_1 differs from that of $\overline{M_1}$; for example, for *ARID1B*, head circumference (OFC) is discriminative, but weight is not (Figure 1A).

We next sought methods for selecting HPO terms and deriving useful probabilistic models from them in the likelihood framework. Information content (IC), defined as –log(probability of term),[24] has been combined with genomic frequency and has been used to compare ontology-encoded phenotypes to aid prediction.[8,20] When considering the entire corpus of annotations (to all 13,439 individuals whether diagnosed or not), the least frequently used terms are most informative but describe the fewest individuals. First, annotation frequencies for all terms, whether used directly in annotation or not, were found by propagating all annotations to their parent terms. A set of *informative phenotypic terms* (IPTs) was identified as follows: starting with the top-level terms for phenotypic abnormality that distinguish disorders of the major organ systems and developmental processes, each was expanded into a set of child terms that met minimal and maximal annotation frequency criteria across the entire cohort (2% and 10% respectively) (Figure 1C). Working in a top-down manner to preferentially select terms that balance IC with generality in the ontology graph, child terms with usage above the upper limit were expanded, and those within lower and upper limits were retained in the IPT set. As IPTs were identified by expanding terms under phenotypic abnormality, an IPT might be found by multiple paths. The IPT data structure was organized so each IPT occurred under a single top-level term, and where an IPT had a parent that was also an IPT, the annotation matrix was modified so annotation to the parent IPT was deleted for individuals with the child term (Figures 1D and 1E). This procedure and the scripts that implement it are described in more detail in the tutorial in File S1. The frequency of use of IPTs was found from the modified annotation matrix in a computationally efficient manner. Examples of informative phenotypic terms included Mild, Moderate, and Severe global developmental delay (HP:0011342, HP:0011343, HP:0011344) organized under top-level IPT Abnormality of the nervous system (HP:0000707) (Table 1, File S2).

This procedure uses term frequencies from annotations to all individuals irrespective of their diagnosis, seeking terms with moderate IC across the dataset. The resulting 157 terms were used as features in classifiers for all genes we modeled: their frequency of use was found from the modified annotation matrix for IPTs when it was factored by diagnosis (supplemental information).

Measures from information retrieval including term frequency (TF) and inverse document frequency (IDF) have previously been adopted for the selection of relevant HPO terms.[25] To compare our set of IPTs with those from an information theoretic (TF IDF IC) approach, we computed these measures for our dataset and examined the position of our IPTs in a ranking of terms per gene (Figure 2A). IPTs seldom ranked highest by TF IDF IC and can rank rather lowly, so they would not likely be selected by such a method. Terms ranking highly by TF IDF IC had considerably higher information content than IPTs, but considering the top 10 such terms, the number of terms per individual was low (Figure 2B). Applying the same ranking procedure to the terms that define an HPO gene model[1,26](see web resources), we observed a more uniform ranking of HPO terms, where some terms ranked highly and others very lowly (Figure S1). We conclude that our approach uncovers a set of terms that are unlikely to have been selected by conventional metrics.

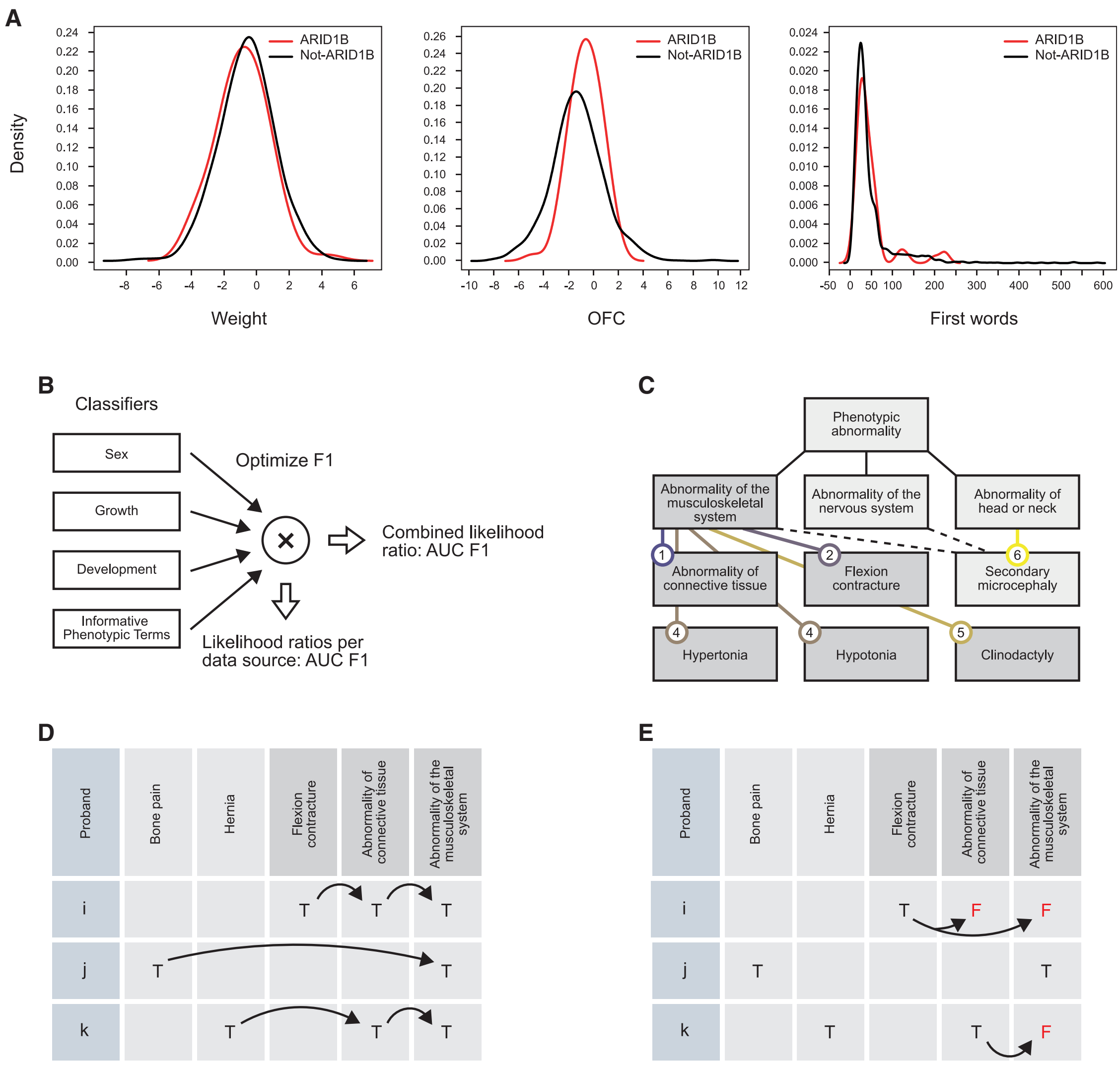

Figure 1. Integrating probabilistic models for nominal, continuous, and ontology annotation data
(A) Continuous data for growth and development is modeled by a smoothed kernel density.
(B) Classifiers for sex, growth, development, and HPO terms are constructed and evaluated individually, and they are combined by optimally weighting their output likelihoods.
(C) All top-level HPO phenotypes (immediately below *Phenotypic abnormality*) are considered informative phenotypic terms (IPTs). Dependent on their annotation frequency across the cohort, child terms are selected as IPTs (250–1500 uses) or further expanded (>1,500 uses). IPTs such as *Secondary microcephaly* that can be selected by multiple paths are included under one top-level IPT only. The number of HPO levels a child term is below its top-level term is given at the end of the arc.
(D) Annotations are propagated from child to parent terms (arrows) to expand the annotation matrix according to the HPO structure.
(E) Annotations to IPTs that arise from propagation from an IPT are removed: individual *i* has *Flexion contracture* but not parents of this term, whereas individual *j* has *Abnormality of the musculoskeletal system* among the annotations to informative terms, as *Bone pain* is not selected as informative.

As summary statistics, we report the F1 score, being the harmonic mean of precision and recall, as a measure of the accuracy of decision-making, and area under the curve (AUC) as a measure of the rank of true positives irrespective of decision threshold across the entire dataset. The individual classifier outputs were combined by a set of weights, found per gene, that optimized F1 from the four input likelihoods plus an additional constant representing the prior. We discuss F1 and AUC from testing on the training data, as indicative of the potential of phenotype modeling, and results from a leave-one-out cross-validation that better controls for model overfitting.

Beginning with the results from testing on the training data, the IPT-based HPO classifier had the best performance in decision-making for most genes (Figure 3A) with F1 scores of up to 0.46 when testing on training data. Exceptions were apparent; for example, growth was a better predictor for *NSD1*, while development was a better predictor for *GRIN2B* (Sotos syndrome MIM: 117550; GRIN2B MIM: 138252). Genes with larger numbers of individuals tended to score well when testing on training data: Pearson correlation between F1 from the HPO classifier and the number of individuals per gene was 0.61 (0.51 in cross-validation; $p < 1e-5$) indicating that performance

Table 1. Examples of informative phenotypic terms

Abnormality of the nervous system (HP:0000707)	Abnormality of the cardiovascular system (HP:0001626)	Abnormality of limbs (HP:0040064)
Mild global developmental delay (HP:0011342)	Abnormal heart morphology (HP:0001627)	Abnormal 5th finger morphology (HP:0004207)
Moderate global developmental delay (HP:0011343)	Abnormality of the vasculature (HP:0002597)	Abnormal thumb morphology (HP:0001172)
Severe global developmental delay (HP:0011344)	Abnormal cardiovascular system physiology (HP:0011025)	Abnormal fingertip morphology (HP:0001211)

was positively influenced by the number of individuals. In the interpretation of F1 scores, it should be noted that the prior probabilities $P(M_i)$ derived from the number of individuals range from 1/150 to 1/19, whereas the alternative hypotheses are much more probable (95/100 to 99/100). Consequently, the evidence from the data commonly failed to outweigh the prior, so precision or recall was 0, and F1 could not be calculated. We found the classification results were insensitive to the annotation frequency criteria used to select IPTs (supplemental information).

The best F1 scores from growth data alone were from *NSD1*, *PTEN*, and *DNMT3A* (Figures 3A, S2, and S3) with 20, 12, and 14 individuals, respectively (top three on cross-validation and top five when testing on training data). Turning to developmental milestones, the best F1 scores were from *SCN8A*, *FOXG1*, and *GRIN2B* (Figures 3A, S2, and S3) with 14, 21, and 25 individuals, respectively (top three by cross-validation and in the top eight testing on training data).

The best predicted genes from HPO annotations were *PTPN11*, *KMT2A*, and *ARID1B* with 25, 69, and 71 individuals, respectively (top three on leave-one-out cross-validation, top five when testing on training data). The likelihoods of individual IPTs can be examined for each individual and provide potentially useful diagnostic information to a clinician. We list the three most likely and three least likely *KMT2A* diagnoses according to phenotype modeling to show the balance of HPO terms for and against and, in the final case, the positive contribution from growth outweighed by the negative contribution

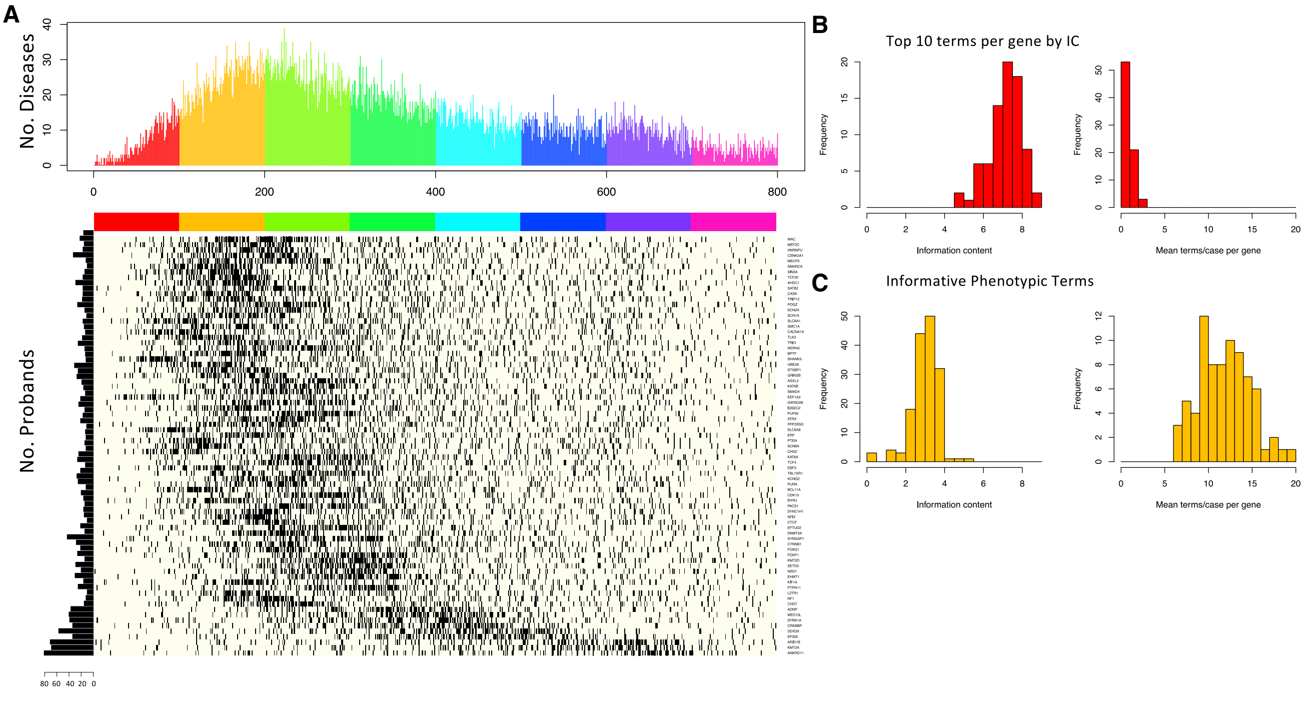

Figure 2. Informative phenotypic terms differ from the highest scoring terms by information theoretic criterion
(A) Heatmap showing for each gene (row) the occurrence of an IPT in a ranking of HPO terms by TF IDF IC. HPO terms are ordered left to right by decreasing TF IDF IC. Top panel shows the number of diseases for which an informative term is found in rank *i* (from 1 to 800), and colors indicate scale, where each covers 100 positions. Although occurring toward the top of the 2,634 terms for which this measure can be calculated, IPTs are seldom in the top 50, and genes such as *ANKRD11*, *ARID1B*, and *KMT2A* in the bottom rows rank 400 and below.
(B) Histograms of mean term IC (left) and mean number of individuals per term (right) when selecting the top 10 terms scored by TF IDF IC per gene.
(C) Histograms of term IC (left) and number of individuals per term (right) for the 157 IPTs.

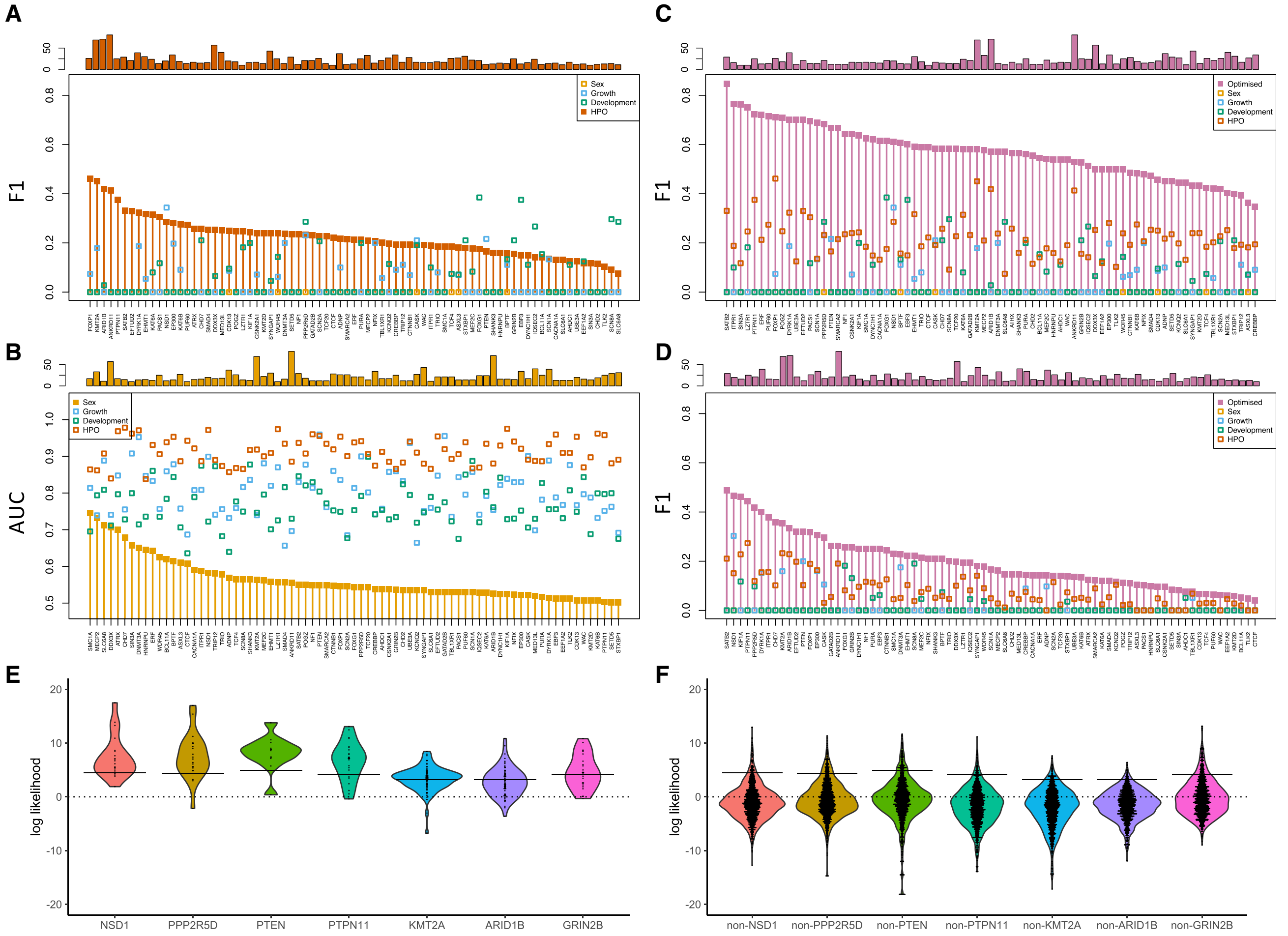

Figure 3. Integrating multiple phenotypic models improves classification
(A) F1 per gene from sex, growth, development, and HPO classifiers (all values plotted at the same x coordinate). Vertical bars and filled symbols highlight the HPO classifier performance. Number of individuals per gene (upper).
(B) AUC per gene highlighting sex.
(C) F1 per gene highlighting the optimized score.
(D) F1 per gene highlighting the optimized score when combining likelihoods from cross-validation.
(E) Violin plots of likelihood ratios from HPO terms for selected genes (symbols are individuals). Likelihoods are shown for a prior of 0.5 to give a common zero reference line for all models. The per-gene prior is shown by the horizontal bar. The HPO model may represent a consensus for a majority of individuals, yet the model may not be sufficient to give a diagnosis for some individuals (*KMT2A*, *ARID1B*). Alternatively, the phenotypic spectrum may be broad (*NSD1*, *PPP2R5D*) and correctly diagnose all but a few cases.
(F) Violin plots for the gene models in (E) applied to individuals whose diagnosis differs. A log likelihood above the per-gene prior is a false positive.

from HPO terms (Table 2). Of note, the top three cases are pathogenic frameshifts, whereas the bottom three are likely pathogenic missense variants.

As would be expected, sex alone was a poor predictor, but this attribute has information by AUC for three genes: *SMC1A*, *MECP2*, and *DDX3X* (Figure 3B). A strong female bias is known for these X-linked syndromes (OMIM MIM: 300040; MIM: 300005; DDX3X MIM: 300160).

Performance measures from testing on the training data (the apparent error) are expected to be optimistic estimates of the true rates. In contrast, the estimates from leave-one-out cross-validation are believed to have less bias and more variance. To further explore issues of cross-validation, we performed a .632[27] bootstrap cross-validation that combined the apparent error with that from held out samples in a bootstrap procedure. Due to the imbalance of the classes, the method could only be applied to the recall of the class of interest, which was shown to be intermediate between the apparent and leave-one-out values as would be expected (supplemental information).

We next optimized the F1 score for each gene by combining the likelihood ratios for each data type using five weights w_0–w_4 (Equation 2). For a given set of weights, a value from Equation 2 greater than 0 is a classification to the gene, from which true and false positives can be determined and F1 calculated.

$$w_0\left(Ps(M_1)/Ps(\overline{M_1})\right)^{w_1}\left(Pg(M_1)/Pg(\overline{M_1})\right)^{w_2}\left(Pd(M_1)/Pd(\overline{M_1})\right)^{w_3}\left(Ph(M_1)/Ph(\overline{M_1})\right)^{w_4} \quad \text{(Equation 2)}$$

Table 2. Clinical evidence supporting or opposing the diagnosis of *KMT2A* in individuals with a pathogenic *de novo* variant

DECIPHER ID	Growth	Dev.	HPO	Total[a]	Informative phenotypic terms supporting *KMT2A*	Informative phenotypic terms opposing *KMT2A*
295774	2.5	2.0	18.2	12.9	Abnormal size of the palpebral fissures (1.4) Short stature (1) Intellectual disability mild (0.45) Abnormality of the endocrine system (0.44)	—
258419	5.0	0.8	16.2	12.2	Short stature (1) Abnormality of the endocrine system (0.44) Cognitive impairment (0.39)	—
294226	2.3	0.9	18.2	11.7	Abnormal size of the palpebral fissures (1.4) Short stature (1) Abnormal hair quantity (0.95) Abnormality of upper lip (0.41)	Abnormality of the nervous system (−0.011)
304702	−1.5	1.4	−1.3	−11.1	Epicanthus (0.88) Syndactyly (0.64) Abnormality of the genital system (0.23) Abnormality of prenatal development or birth (0.22)	Abnormality of the musculoskeletal system (−0.61) Abnormality of the forehead (−0.41) Abnormality of lower lip (−0.39) Abnormality of the fontanelles or cranial sutures (−0.37) Thick vermilion border (−0.37) Moderate global developmental delay (−0.34) Abnormality of toe (−0.017)
273901	−0.6	−0.4	−7.9	−19.3	Abnormal hair quantity (0.95) Abdominal symptom (0.45) Abnormality of eye movement (0.038)	Involuntary movements (−1.4) Generalized-onset seizure (−1.3) Abnormality of coordination (−0.82) Gait disturbance (−0.64) Dialeptic seizure (−0.46) Non-motor seizure (−0.28) Hypotonia (−0.22) Sleep disturbance (−0.2) Abnormality of the respiratory system (−0.041) Abnormality of the immune system (−0.02)
305957	5.7	−0.4	−17.6	−22.1	—	Abnormal ear physiology (−1.2) Abnormality of calvarial morphology (−1) Abnormality of skin pigmentation (−0.91) Abnormal emotion/affect behavior (−0.62) Abnormality of the musculoskeletal system (−0.61) Abnormality of the middle ear (−0.28) Localized skin lesion (−0.075)

[a]Values are log likelihood ratios. Note that the total includes values not listed here.

In Equation 2, $P(D|M)$ is abbreviated to $Ps(M)$ for sex, and similarly for growth (Pg), development (Pd), and HPO (Ph). Simulated annealing implemented in the R package GenSA was used to find the optimal weights (web resources).

For all genes, optimization improved F1 over any individual data source (Figure 3C), achieving F1 scores greater than 0.7 for 12 genes when testing on training data. Genes with lower numbers of individuals were high in the ranking, indicating that good models can be found for them, but overfitting in the original model training may be at play. The F1 score was generally reduced in leave-one-out cross-validation where 14 genes had F1 0.3–0.5, namely, *ARID1B*, *CHD7*, *DYRK1A*, *EFTUD2*, *EP300*, *FOXP1*, *ITPR1*, *KIF1A*, *KMT2A*, *NSD1*, *PPP2R5D*, *PTEN*, *PTPN11*, and *SATB2* (Figures 3D and S3). Genes with larger numbers of individuals tended to have higher F1 (Pearson correlation 0.27; $p = 0.015$).

We also examined the distribution of likelihoods of a diagnosis (Figure 3E) versus all others (Figure 3F) from HPO gene models. This analysis highlighted a number of genes, including *KMT2A*, where the evidence from the fit to the HPO model did not outweigh the prior for many individuals and hence gave false negatives.

We then asked if an optimized HPO classifier would rival the combined data classifier. Optimal values were found for w_0 and w_4 (for the prior and HPO likelihood) using the same procedure. Using only HPO terms gave 147 fewer true positives (8.5% of the 1,730 individuals) and 2,041 more false positives in total (summing false positives over 77 genes). Per gene, median recall was reduced from 0.57 to 0.5, and median precision from 0.64 to 0.21. The benefits of additional phenotypic information, specifically growth and development data, are clear from these results. As an additional comparison, classifiers based on top terms by TF IDF IC and by disease model were also assessed and found to not perform as well as IPT-based classifiers (supplemental information).

To further investigate the generality of each model in each data type, we assessed growth, development, and HPO models through their contribution to the optimized log likelihood for all individuals for each gene (Figure 4A). This revealed models that worked well in decision-making did not necessarily capture all diagnosed individuals; for example, growth in *NSD1* and development in *GRIN2B* captured distinctive subsets of individuals. To visualize this across all 77 genes, we selected individuals at quartiles 1, 2, and 3 representing poor, typical, and good fits to the gene models (Figure 4B). Where the scaled values were negative, the model contradicted the assigned diagnosis. We found HPO models agreed with the correct

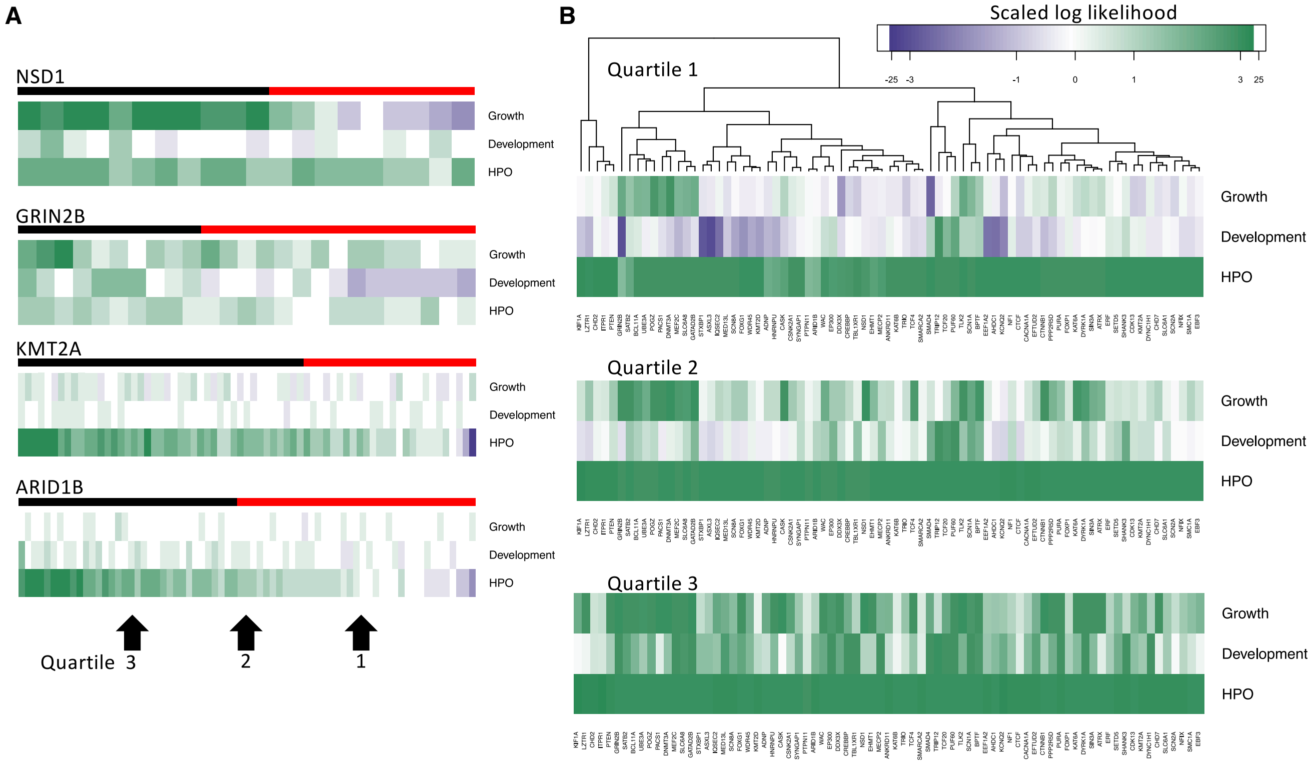

Figure 4. The contribution of data type to diagnosis varies by gene
(A) Heatmaps of log likelihood per individual (column) for each data type (row) for selected genes. Values are scaled by optimization weight and columns ordered right to left from highest to lowest total likelihood (negative values in blue, positive in green). Upper bar shows true positives in black and false negatives in red.
(B) Heatmaps of log likelihood multiplied by optimization weight per gene (column) for each data type (row). Heatmaps show values for individuals at quartiles 1, 2, and 3 successively for each data type. The hierarchical clustering reflects groupings at quartile 1. For example, for *GRIN2B*, the individual with the first quartile score fits the development model poorly (indicated in blue), and the median *GRIN2B* individual has a small negative contribution from development, and the contribution from development is positive by the third quartile. Only positive contributions are found from quartile 3, and HPO has a positive contribution at each quartile. The color scale truncates absolute values above 3 in order to focus on the range −3 to 3.

diagnosis at each quartile. Defining models to generalize well as those in which a positive likelihood ratio is found for the median individual, all but four growth models generalized well. However, development models for 22 genes (29%) failed to generalize by this criterion.

In summary, we found that HPO terms are the best predictor of a correct diagnosis for most genes, but 17% (13/77 genes) were better predicted by growth or development, and prediction from combined data performed better than any individual source. Predictions from combined data also gave notably fewer false positives than prediction from HPO terms alone, as median precision increased to 0.64 from 0.21 when only HPO terms were used. While more individuals are to be preferred when building models, we found gene models with good predictive performance on cross-validation could be built from a relatively small number of individuals ($n \geq 10$). The derivation of likelihood ratios from individual annotations using HPO-encoded disease models of LIRICAL[6] is closest to our approach. However, rather than define HPO models per gene, we began with the extensive database of individual annotations of the DDD study from which we are able to assess HPO term usage in clinical practice irrespective of diagnosis: our disease models are then computed from the observed annotations for each gene.

Bayesian methods are recognized as adding quantitative rigor to the combination of evidence for and against variant pathogenicity in rare disease while making explicit any assumptions regarding strength of evidence and disease prevalence.[28] In addition, they are responsive to changes in the evidence base as new observations are made. We extend the application of this paradigm to phenotypic data making use of the extensive acquisition of growth measures and developmental milestones in addition to HPO terms in the DDD study. This approach could be extended by the incorporation of additional phenotypic data (e.g., images, epigenomic profiles, biochemical assays, etc.) to further improve gene-disease models and make them more applicable to other rare disorders. Although phenotypic models are unlikely to be sufficiently predictive by themselves, particularly for genetically heterogeneous disorders such as DD, they can be used to update posterior probabilities found from genomic analyses of

variant pathogenicity[28] and thus have an important role in increasing the robustness of a diagnosis. This is illustrated for an individual with a missense variant in *NSD1* with weak genetic evidence that could be strengthened to likely pathogenic through the likelihood ratio from HPO terms by the methods reported here.[29]

Quantitative patient data is of importance to clinical interpretation, and we have proved its value in computational disease modeling; however, systematic collection of and computational access to such data is often lacking in health data systems, potentially hindering diagnostic and biological insights.

Data and code availability

The code used to identify informative HPO terms and to run the classification and optimization procedures can be found in the IMPROVE-DD github repository. Sequence and variant-level data and phenotypic data for the DDD study data are available from the European Genome-phenome Archive (EGA; https://www.ebi.ac.uk/ega/) with study ID EGAS00001000775. Clinically interpreted variants and associated phenotypes from the DDD study are available through DECIPHER (https://www.deciphergenomics.org/).

Supplemental information

Supplemental information can be found online at https://doi.org/10.1016/j.xhgg.2022.100162.

Acknowledgments

The DDD study presents independent research commissioned by the Health Innovation Challenge Fund (grant number HICF-1009-003), a parallel funding partnership between Wellcome and the Department of Health, and the Wellcome Sanger Institute (grant number WT098051). The views expressed in this publication are those of the authors and not necessarily those of Wellcome or the Department of Health. The study has UK Research Ethics Committee approval (10/H0305/83, granted by the Cambridge South REC, and GEN/284/12 granted by the Republic of Ireland REC). The research team acknowledges the support of the National Institute for Health Research, through the Comprehensive Clinical Research Network. This study makes use of DECIPHER (https://www.deciphergenomics.org/), which is funded by the Wellcome. H.V.F. is supported by the Wellcome Trust (award 200990/Z/16/Z) "Designing, developing and delivering integrated foundations for genomic medicine." The research team acknowledges the support of the National Institute for Health Research, through the Comprehensive Clinical Research Network. D.R.F. is funded as part of the MRC Human Genetics Unit grant to the University of Edinburgh. C.A.S. and S.A. are supported by MRC Core funding to the MRC Human Genetics Unit (MRC grant MC_UU_00007/16). For the purpose of open access, the authors have applied a CC-BY public copyright license to any author-accepted manuscript version arising from this submission.

Declaration of interests

M.E.H. is a co-founder, consultant, and non-executive director of Congenica Ltd.

Received: May 25, 2022
Accepted: November 22, 2022

Web resources

DECIPHER, https://www.deciphergenomics.org/

EGA, https://www.ebi.ac.uk/ega/

genSA, https://CRAN.R-project.org/package=GenSA.

HPO https://hpo.jax.org/app/

HPO, Gene Models http://purl.obolibrary.org/obo/hp/hpoa/genes_to_phenotype.txt.

IMPROVE-DD scripts, https://github.com/Stuart-Aitken/IMPROVE-DD.

Naivebayes, https://CRAN.R-project.org/package=naivebayes

References

1. Köhler, S., Gargano, M., Matentzoglu, N., Carmody, L.C., Lewis-Smith, D., Vasilevsky, N.A., Danis, D., Balagura, G., Baynam, G., Brower, A.M., et al. (2021). The human phenotype ontology in 2021. Nucleic Acids Res. *49*, D1207–D1217. https://doi.org/10.1093/nar/gkaa1043.
2. Aitken, S., Firth, H.V., McRae, J., Halachev, M., Kini, U., Parker, M.J., Lees, M.M., Lachlan, K., Sarkar, A., Joss, S., et al. (2019). Finding diagnostically useful patterns in quantitative phenotypic data. Am. J. Hum. Genet. *105*, 933–946. https://doi.org/10.1016/j.ajhg.2019.09.015.
3. Ferry, Q., Steinberg, J., Webber, C., FitzPatrick, D.R., Ponting, C.P., Zisserman, A., and Nellåker, C. (2014). Diagnostically relevant facial gestalt information from ordinary photos. Elife *3*, e02020. https://doi.org/10.7554/eLife.02020.
4. FitzPatrick, D.R., and Firth, H.V. (2020). Genomically aided diagnosis of severe developmental disorders. Annu. Rev. Genomics Hum. Genet. *21*, 327–349. https://doi.org/10.1146/annurev-genom-120919-082329.
5. Köhler, S., Øien, N.C., Buske, O.J., Groza, T., Jacobsen, J.O.B., McNamara, C., Vasilevsky, N., Carmody, L.C., Gourdine, J.P., Gargano, M., et al. (2019). Encoding clinical data with the human phenotype ontology for computational differential diagnostics. Curr. Protoc. Hum. Genet. *103*, e92. https://doi.org/10.1002/cphg.92.
6. Robinson, P.N., Ravanmehr, V., Jacobsen, J.O.B., Danis, D., Zhang, X.A., Carmody, L.C., Gargano, M.A., Thaxton, C.L., UNC Biocuration Core, and Karlebach, G., et al. (2020). Interpretable clinical genomics with a likelihood ratio paradigm. Am. J. Hum. Genet. *107*, 403–417. https://doi.org/10.1016/j.ajhg.2020.06.021.
7. Li, Z., Zhang, F., Wang, Y., Qiu, Y., Wu, Y., Lu, Y., Yang, L., Qu, W.J., Wang, H., Zhou, W., and Tian, W. (2019). PhenoPro: a novel toolkit for assisting in the diagnosis of Mendelian disease. Bioinformatics *35*, 3559–3566. https://doi.org/10.1093/bioinformatics/btz100.
8. Li, Q., Zhao, K., Bustamante, C.D., Ma, X., and Wong, W.H. (2019). Xrare: a machine learning method jointly modeling phenotypes and genetic evidence for rare disease diagnosis. Genet. Med. *21*, 2126–2134. https://doi.org/10.1038/s41436-019-0439-8.
9. Xue, H., Peng, J., and Shang, X. (2019). Predicting disease-related phenotypes using an integrated phenotype similarity measurement based on HPO. BMC Syst. Biol. *13*, 34. https://doi.org/10.1186/s12918-019-0697-8.

10. Gao, J., Liu, L., Yao, S., Huang, X., Mamitsuka, H., and Zhu, S. (2019). HPOAnnotator: improving large-scale prediction of HPO annotations by low-rank approximation with HPO semantic similarities and multiple PPI networks. BMC Med. Genomics *12*, 187. https://doi.org/10.1186/s12920-019-0625-1.
11. Akawi, N., McRae, J., Ansari, M., Balasubramanian, M., Blyth, M., Brady, A.F., Clayton, S., Cole, T., Deshpande, C., Fitzgerald, T.W., et al. (2015). Discovery of four recessive developmental disorders using probabilistic genotype and phenotype matching among 4, 125 families. Nat. Genet. *47*, 1363–1369. https://doi.org/10.1038/ng.3410.
12. Javed, A., Agrawal, S., and Ng, P.C. (2014). Phen-Gen: combining phenotype and genotype to analyze rare disorders. Nat. Methods *11*, 935–937. https://doi.org/10.1038/nmeth.3046.
13. Kulmanov, M., and Hoehndorf, R. (2020). DeepPheno: predicting single gene loss-of-function phenotypes using an ontology-aware hierarchical classifier. PLoS Comput. Biol. *16*, e1008453. https://doi.org/10.1371/journal.pcbi.1008453.
14. Boudellioua, I., Kulmanov, M., Schofield, P.N., Gkoutos, G.V., and Hoehndorf, R. (2019). DeepPVP: phenotype-based prioritization of causative variants using deep learning. BMC Bioinf. *20*, 65. https://doi.org/10.1186/s12859-019-2633-8.
15. Pontikos, N., Yu, J., Moghul, I., Withington, L., Blanco-Kelly, F., Vulliamy, T., Wong, T.L.E., Murphy, C., Cipriani, V., Fiorentino, A., et al. (2017). Phenopolis: an open platform for harmonization and analysis of genetic and phenotypic data. Bioinformatics *33*, 2421–2423. https://doi.org/10.1093/bioinformatics/btx147.
16. Köhler, S., Schulz, M.H., Krawitz, P., Bauer, S., Dölken, S., Ott, C.E., Mundlos, C., Horn, D., Mundlos, S., and Robinson, P.N. (2009). Clinical diagnostics in human genetics with semantic similarity searches in ontologies. Am. J. Hum. Genet. *85*, 457–464. https://doi.org/10.1016/j.ajhg.2009.09.003.
17. Bone, W.P., Washington, N.L., Buske, O.J., Adams, D.R., Davis, J., Draper, D., Flynn, E.D., Girdea, M., Godfrey, R., Golas, G., et al. (2016). Computational evaluation of exome sequence data using human and model organism phenotypes improves diagnostic efficiency. Genet. Med. *18*, 608–617. https://doi.org/10.1038/gim.2015.137.
18. Yang, J., Dong, C., Duan, H., Shu, Q., and Li, H. (2021). RDmap: a map for exploring rare diseases. Orphanet J. Rare Dis. *16*, 101. https://doi.org/10.1186/s13023-021-01741-4.
19. Deciphering Developmental Disorders Study, Gerety, S.S., Jones, W.D., van Kogelenberg, M., King, D.A., McRae, J., Morley, K.I., Parthiban, V., Al-Turki, S., Ambridge, K., et al. (2015). Large-scale discovery of novel genetic causes of developmental disorders. Nature *519*, 223–228. https://doi.org/10.1038/nature14135.
20. Deciphering Developmental Disorders Study, Clayton, S., Fitzgerald, T.W., Kaplanis, J., Prigmore, E., Rajan, D., Sifrim, A., Aitken, S., Akawi, N., Alvi, M., et al. (2017). Prevalence and architecture of de novo mutations in developmental disorders. Nature *542*, 433–438. https://doi.org/10.1038/nature21062.
21. Wright, C.F., Fitzgerald, T.W., Jones, W.D., Clayton, S., McRae, J.F., van Kogelenberg, M., King, D.A., Ambridge, K., Barrett, D.M., Bayzetinova, T., et al. (2015). Genetic diagnosis of developmental disorders in the DDD study: a scalable analysis of genome-wide research data. Lancet *385*, 1305–1314.
22. Firth, H.V., Wright, C.F.; and DDD Study (2011). The deciphering developmental disorders (DDD) study. Dev. Med. Child Neurol. *53*, 702–703. https://doi.org/10.1111/j.1469-8749.2011.04032.x.
23. Firth, H.V., Richards, S.M., Bevan, A.P., Clayton, S., Corpas, M., Rajan, D., Van Vooren, S., Moreau, Y., Pettett, R.M., and Carter, N.P. (2009). DECIPHER: database of chromosomal imbalance and phenotype in humans using ensembl resources. Am. J. Hum. Genet. *84*, 524–533. https://doi.org/10.1016/j.ajhg.2009.03.010.
24. Resnik, P. (1999). Semantic similarity in a taxonomy: an information-based measure and its application to problems of ambiguity in natural language. J. Artif. Intell. Res. *11*, 95–130.
25. Groza, T., Köhler, S., Moldenhauer, D., Vasilevsky, N., Baynam, G., Zemojtel, T., Schriml, L.M., Kibbe, W.A., Schofield, P.N., Beck, T., et al. (2015). The human phenotype ontology: semantic unification of common and rare disease. Am. J. Hum. Genet. *97*, 111–124. https://doi.org/10.1016/j.ajhg.2015.05.020.
26. Köhler, S., Carmody, L., Vasilevsky, N., Jacobsen, J.O.B., Danis, D., Gourdine, J.-P., Gargano, M., Harris, N.L., Matentzoglu, N., McMurry, J.A., et al. (2019). Expansion of the human phenotype ontology (HPO) knowledge base and resources. Nucleic Acids Res. *47*, D1018–D1027. https://doi.org/10.1093/nar/gky1105.
27. Efron, B. (1983). Estimating the error rate of a prediction rule: improvement on cross-validation. J. Am. Stat. Assoc. *78*, 316–331. https://doi.org/10.2307/2288636.
28. Tavtigian, S.V., Greenblatt, M.S., Harrison, S.M., Nussbaum, R.L., Prabhu, S.A., Boucher, K.M., Biesecker, L.G.; and ClinGen Sequence Variant Interpretation Working Group ClinGen SVI (2018). Modeling the ACMG/AMP variant classification guidelines as a Bayesian classification framework. Genet. Med. *20*, 1054–1060. https://doi.org/10.1038/gim.2017.210.
29. Wright, C.F., Campbell, P., Eberhardt, R.Y., Aitken, S., Perrett, D., Brent, S., Danecek, P., Gardner, E.J., Chundru, V.K., Lindsay, S.J., et al. (2022). Optimising diagnostic yield in highly penetrant genomic disease. Preprint at medRxiv. https://doi.org/10.1101/2022.07.25.22278008.

HGGA, Volume *4*

Supplemental information

IMPROVE-DD: Integrating multiple phenotype resources optimizes variant evaluation in genetically determined developmental disorders

Stuart Aitken, Helen V. Firth, Caroline F. Wright, Matthew E. Hurles, David R. FitzPatrick, and Colin A. Semple

Tutorial on creating sets of Informative Phenotypic Terms

Finding IPTs in the DDD database

Informative Phenotypic Terms (IPTs) were derived from a binary matrix of terms annotated to all DDD individuals (individuals in rows, HPO terms in columns) and an OBO format version of the HPO ontology (that of 02/08/2021). An extended matrix of propagated annotations was then created (direct annotations were propagated to parent terms using the ontology structure). The usage of HPO terms was then found for all HPO terms making no distinction between direct and inferred use.
Beginning with top level phenotypic terms (those under “Phenotypic abnormality” HP:0000118), all such terms were included as IPTs, and each was descended in turn to select subterms meeting the criterion of use in the extended annotation matrix (above 250 and below 1500 uses in the entire DDD dataset of 13439 individuals). Subterms with usage above the upper threshold were further expanded, and the criterion applied at that level. As there can be multiple paths to a term from the top level, an IPT can be found under multiple top level terms – these were detected and the IPT retained under a single top level term (in a list of lists data structure). The final step was to detect IPTs that, through the ontology structure, had parent terms that were also IPTs. To remove the resulting correlation in annotation of the parent to the child, the extended annotation matrix was modified to remove the annotation to the parent term for individuals with an annotation to the child IPT in question. This modified matrix was used to compute term frequencies in gene models.
The procedure does not depend on any specific version of HPO. The version of HPO specified can be found here: https://bioportal.bioontology.org/ontologies/HP

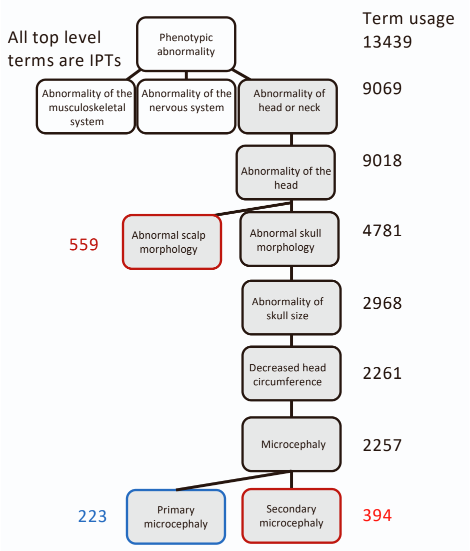

HPO terms were expanded (black) or retained as IPTs (red) according to usage.

Running the resource creation scripts

The following steps are run once for a database of HPO annotations made to individuals, generating a matrix that is used by the HPO classifier. This procedure does not require individuals to have diagnoses (diagnoses are considered in model learning and classification).
Implementing the steps described above, the IMPROVE_resource.r code begins by loading an OBO format of the HPO ontology using the ontoCAT R library (line 27).
In place of the DDD phenotype data (which is available on request), a matrix of individual annotations *hpo* is created from the data in *pheno* (line 67) after finding all terms used in annotation *allHPO*. The following steps can be run on data from another database providing the individual to HPO annotation is expressed as a binary matrix (individuals in rows, HPO terms in columns).
The expanded matrix *hpoe* is created from *hpo* using the *expandToParents()* method of the ontoCAT library (line 101). The entire set of expanded terms (*allHPOExpanded*) is found in a preliminary step in order to define *hpoe*.
The data structure *informativeSpecificTerms* is initialised from terms under HP:0000118 (“Phenotypic abnormality”) and each top level term descended to identify terms meeting usage < *upper_annotation_threshold* and >= *retain_threshold* (lines 126 onwards). In the DDD data, we found 10% and 2% of total annotations were suitable thresholds.
As there can be multiple paths to an IPT, multiple instances may occur in *informativeSpecificTerms*. These are detected by tabluating terms and removing second and third occurrences to create *informativePhenotypicTerms*. This was performed intially after manual inspection (line 196) but is now automated (line 154).
The method *getDuplicatesAndParents()* returns information on any child-parent edges within *informativePhenotypicTerms* (line 255). A modified annotation matrix *hpou* is created from *hpoe* by removing parent annotation in individuals where there is an annotation to the child (lines 270). The *hpou* matrix is the resource used in the classifier. The HPO itself is not needed in classification

Methods

Classification and optimisation

Classifiers for nominal and continuous data were implemented using the naivebayes R package (Web Resources). For all growth and development data, the bandwidth of the nrd0 kernel to was increased to 1.5 to remove overfitting.
Optimisation was performed using the genSA R package (Web Resources). The inputs to optimisation were the likelihood ratios calculated by the models for each data source for all individuals. As the prior had been accounted for in all four cases, the priors were balanced to equality between the gene model and the alternative (as in **Figure 3E** and **3F**) and w_0 included in the optimisation as replacement. Optimisation maximised F1 by combining the likelihood values through the five weights as defined in equation (2).
A sensitivity analysis of parameter values around the optimal (replacing all parameters in each of 1000 iterations by sample from a normal distribution mean equal to the optimal, sd = 1/10 |optimal|) resulted in F1 values narrowly distributed around a reduced mean (**Figure S5**). The relatively small reduction in F1 indicates that non-optimal parameters cause the mis-classification of only a small number of cases.

Term selection

Annotations to HPO terms directly assigned to probands (4182 terms were used across 13439 individuals with a median of 6 terms per proband) were propagated to all parent terms, expanding the annotation to 5153 terms with a median of 40 terms per proband.
157 informative phenotypic terms were selected according to usage as decribed in the main text (**Figure 1D** and **1E**) with a median use of 10 terms per proband. Two alternative thresholds for term inclusion were explored: i. above 500 and below 3000 annotations resulting in 79 terms; and ii. above 125 and below 750 annotations resulting in 268 terms. Recall that the thresholds we adopted were above 250 and below 1500 annotations which gave 157 terms. The alternatives double and halve these thresholds respectively. The thresholds we selected give significantly better AUCs than the smaller set of 79 terms (p= 5.6e-06) although the average effect is small (0.03). There is no statistical difference between AUCs from 268 terms and 157 terms. Scatter plots of these results are now presented (**Figure S6)**. Consequently, we concluded that the choice of thresholds was not critical.

Term probabilities

Gene models were defined by the probabilities of the 157 IPTs in the annotations to diagnosed individuals *D*. The probability of each *IPT* in *D* was given by the m-estimate:

$$P(IPT) = {A(IPT, D) + 1}\Big/{\left(\sum_{t=1}^{t=157} A(t, D)\right) + 100}$$

where the term usage $A(t,D)$ was calculated from the modified annotation matrix as the sum over *t* (column in matrix) for probands (rows) assigned to *D*. The denominator normalises the counts to a specific term by the total annotations made to the 157 IPTs plus a number representing the vocabulary size.
When computing the probability of a set of IPT annotations in a case C_i for a given gene model, the product of probabilities was scaled to adjust for the number of IPTs used in this case. Where C_i had m IPTs the geometric mean probability was raised to the power of 10 (the median number of IPTs per case):

$$P(C_i\ GeneModel) = \prod_{j=1}^{m} P(IPTj)^{10/m}$$

This scaling made the resulting probabilities comparable across probands.
To investigate the impact of alternative term selection strategies, classifiers based on terms selected by TF IDF IC, or from HPO disease models were evaluated. In both cases, the 50 most informative terms per gene were selected (disease model terms were ordered by TF IDF IC) and term probability was based on term frequency in disease with Laplace smoothing:

$$P(HPO) = {A(HPO, D) + 1}\Big/{(|D| + 2)}$$

AUC and F1 were typically lower in gene-specific classifiers than for IPT classifiers when testing on training data (**Figure S4**). We found the occurrence of an HPO term of the gene model in a case to essentially guarantee classification to that model: Mean recall was high (0.79) showing that DDD cases indeed matched these disease models but mean precision was low (0.03). Even though we

selected terms with the highest IC per gene, they occurred in other diagnoses giving many false positives.

.632 bootstrap cross-validation

Performance measures are expected to be optimistic estimates of the true rates when testing on the training data, in contrast, the estimates from leave-one-out cross-validation are believed to have less bias and more variance. The error rate from testing on the training data, known as the apparent error, can be combined with the error from bootstrap resampling (where the error will be a pessimistic estimate to a known degree) to get a more balanced estimate using the .632 method due to Efron (1983). We derived bootstrap estimates for recall based on 17200 samples (ten times the dataset size) for samples absent from the bootstrapped data (sampling with replacement). As there was a large imbalance between the classes in each classification task we used the approach to estimate *recall* of the true positives for gene only, rather than include the much larger number of true negatives in the measure *accuracy*. The results of the .632 cross validation (**Figure S6**) show for HPO data alone, .632 values of recall per gene are mostly intermediate between the apparent error and leave-one-out estimates as would be expected. For the growth and development classification tasks there are many cases where recall is zero (as was the case for F1 by both cross-validation approaches) and again the .632 estimates of recall are mostly intermediate between the other estimates. This analysis indicates leave-one-out may be unduly pessimistic.

Efron, B. (1983) Estimating the Error Rate of a Prediction Rule: Improvement on Cross-Validation *Journal of the American Statistical Association* June 1983, Volume 78, Number 382.

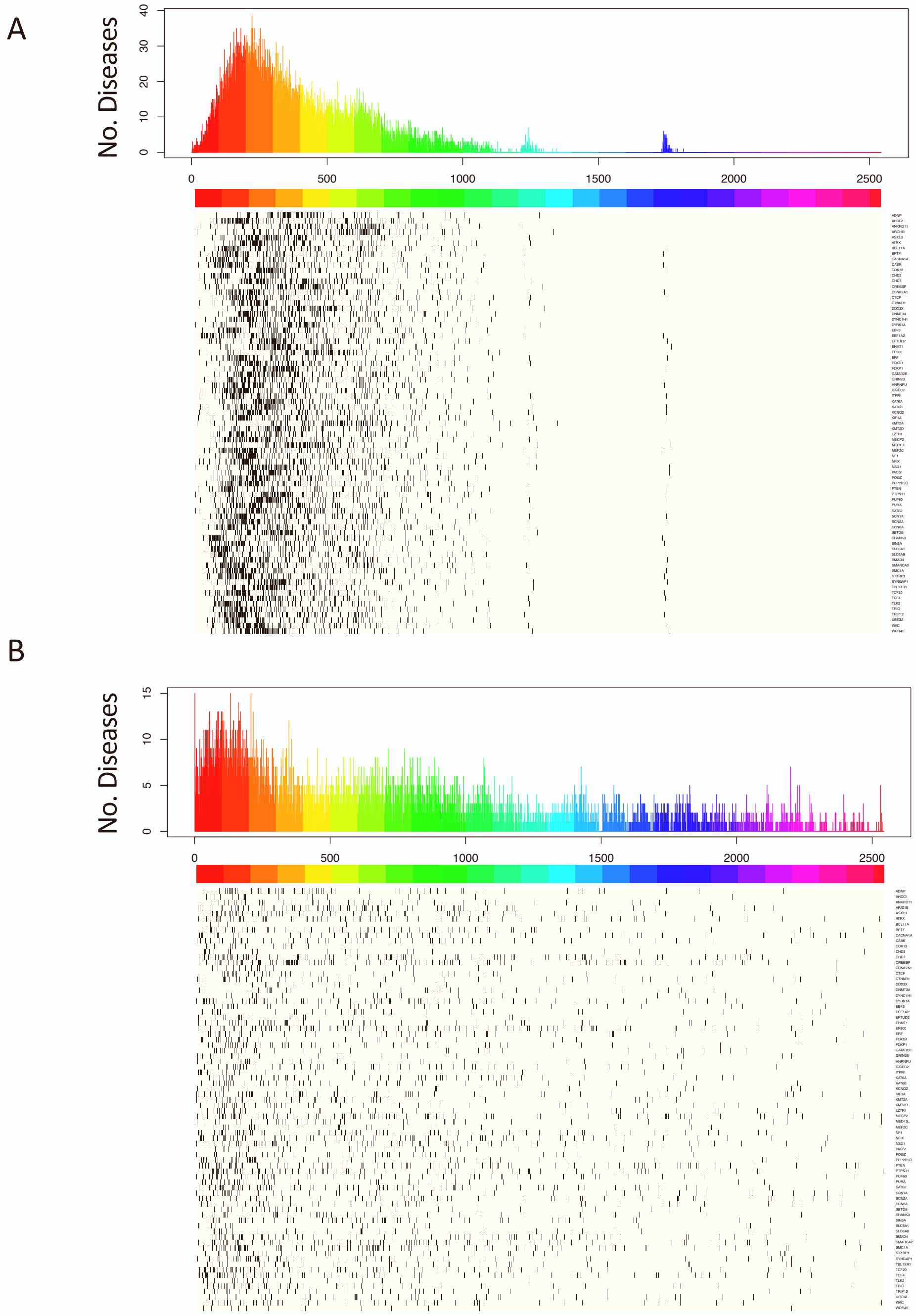

Figure S1. Disease model term ranking

A) Heatmap showing for each gene (row) the occurrence of an informative phenotypic term (IPT) in a ranking of all HPO terms by TF IDF IC. HPO terms are ordered left to right by decreasing TF IDF IC. Top panel shows the number of diseases for which a disease model term is found in rank i (from 1 to 2500), colours indicate scale, each covers 100 positions. Row order is alphabetical by gene name.

B) Heatmap showing for each gene (row) the occurrence of an term in the disease model for that gene in a ranking of all HPO terms by TF IDF IC.

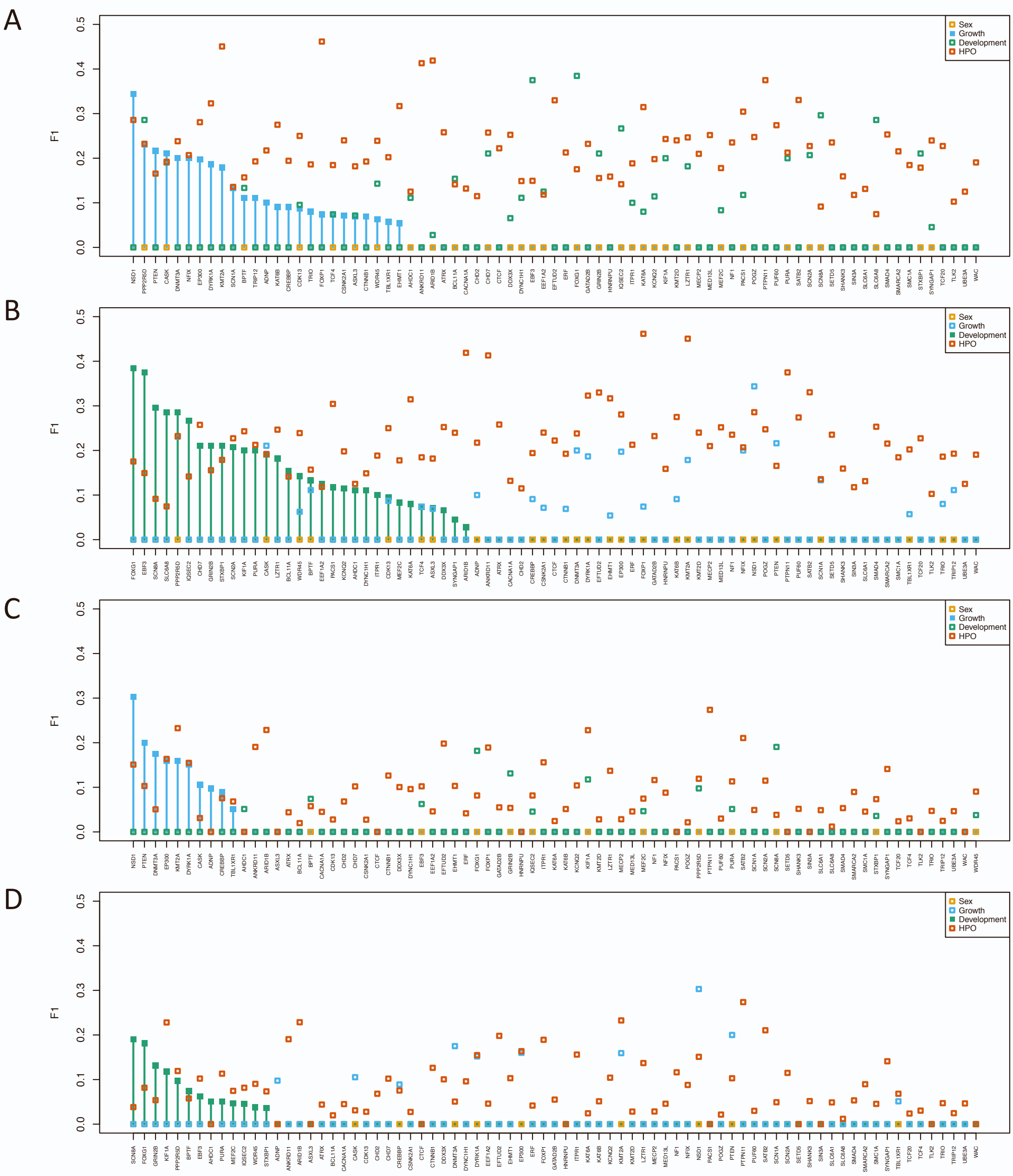

Figure S2. Classifier performance.

A) F1 per gene, testing on training data, ordered by the performance in growth (highlighted by the vertical bars and filled symbols) and B) ordered by performance in development.

C) F1 per gene, from a leave-one-out cross-validation, ordered by the performance in growth (highlighted by the vertical bars and filled symbols), and D) ordered by performance in development.

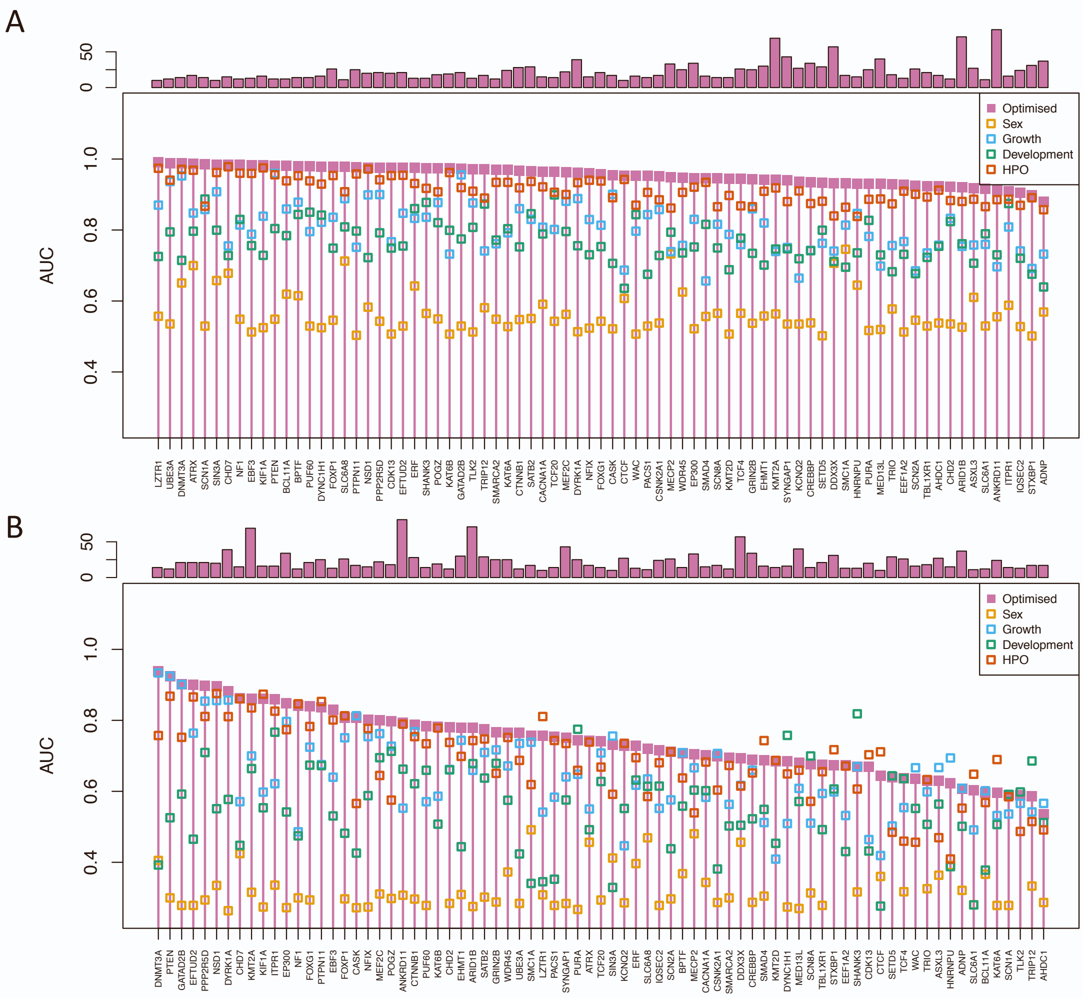

Figure S3. AUC on optimisation.
A) AUC from all data types when testing on training data is shown by symbols, filled symbols and vertical lines highlight the AUC after optimising for F1.
B) AUC from all data types in cross-validation is shown by symbols, filled symbols and vertical lines highlight the AUC after optimising for F1.

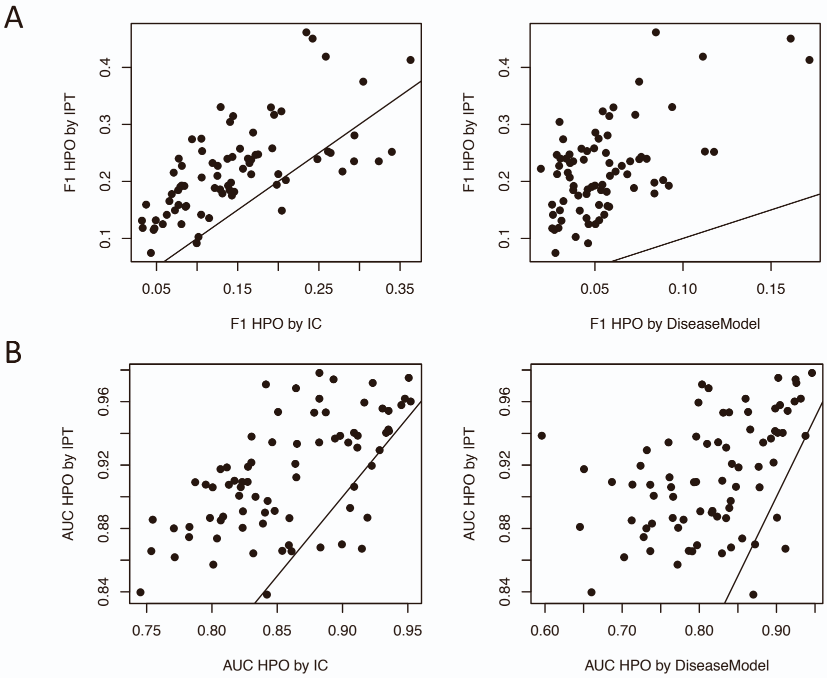

Figure S4. Comparison between IPT and alternative term sets for classification.
A) Scatterplot of F1 from IPTs against F1 from the top 50 terms per gene selected by TF IDF IC (left), and against the top 50 disease model terms per gene selected by TF IDF IC (right). Symbols are genes, lines show y=x.
B) Scatterplot of AUC from IPTs against AUC from the top 50 terms per gene selected by TF IDF IC (left) and against the top 50 disease model terms per gene selected by TF IDF IC (right). Symbols are genes, lines show y=x.

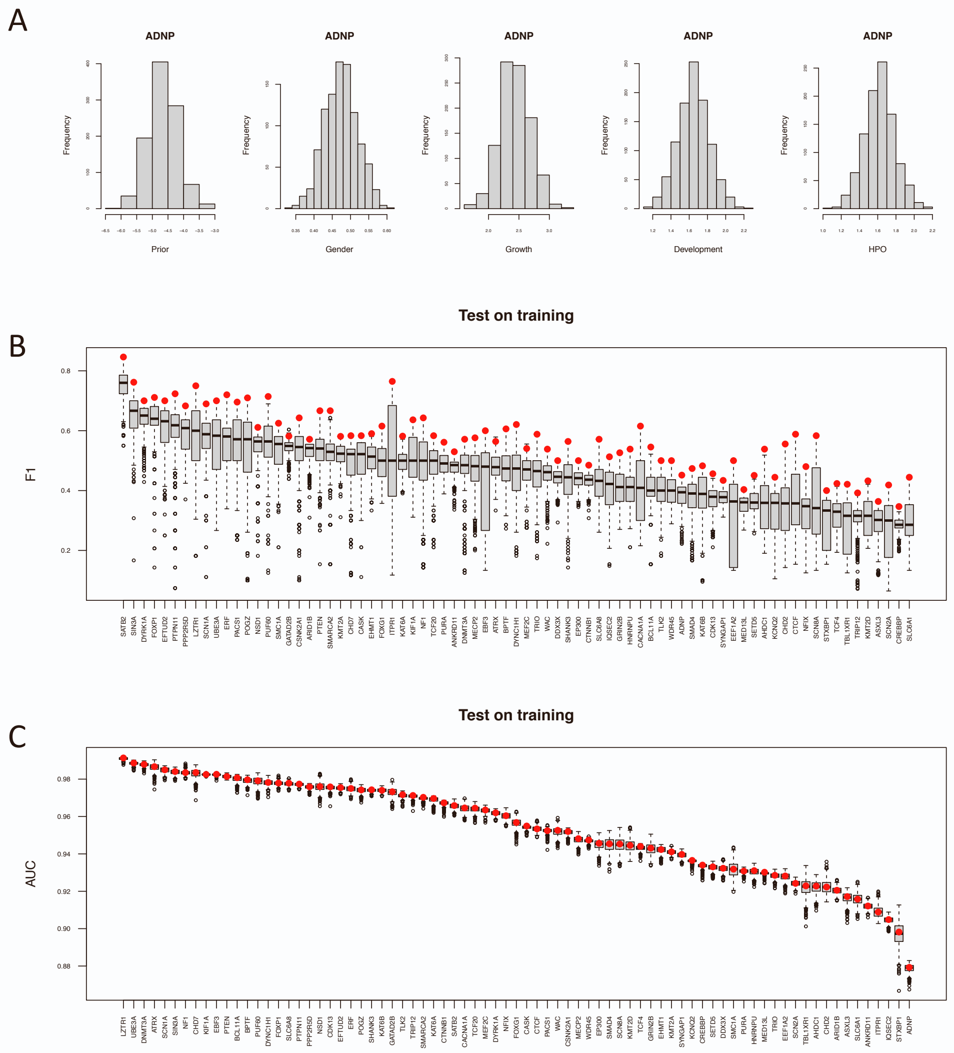

Figure S5. Sensitivity analysis.

A) Histograms of parameter values used in the sensitivity analysis of ADNP.

B) Boxplot of F1 per gene from 1000 random samples of the optimisation weights. F1 from optimal weights in red.

C) Boxplot of AUC per gene from 1000 random samples of the optimisation weights. AUC from optimal weights in red.

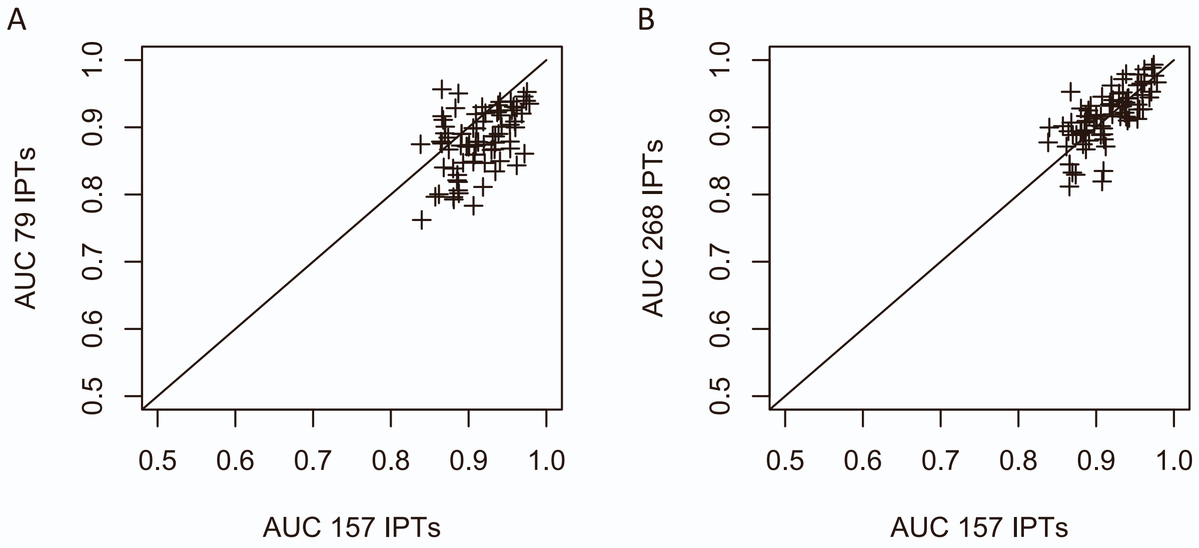

Figure S6. Comparison of alternative thresholds for IPT selection shows AUC per gene is insensitive to parameter choice

A) AUC computed from 79 IPTs selected from HPO terms with usage 500 to 3000 compared with 157 IPTs with usage 250 to 1500. Symbols are genes.

B) AUC computed from 268 IPTs selected from HPO terms with usage 125 to 750 compared with 157 IPTs with usage 250 to 1500. Symbols are genes.

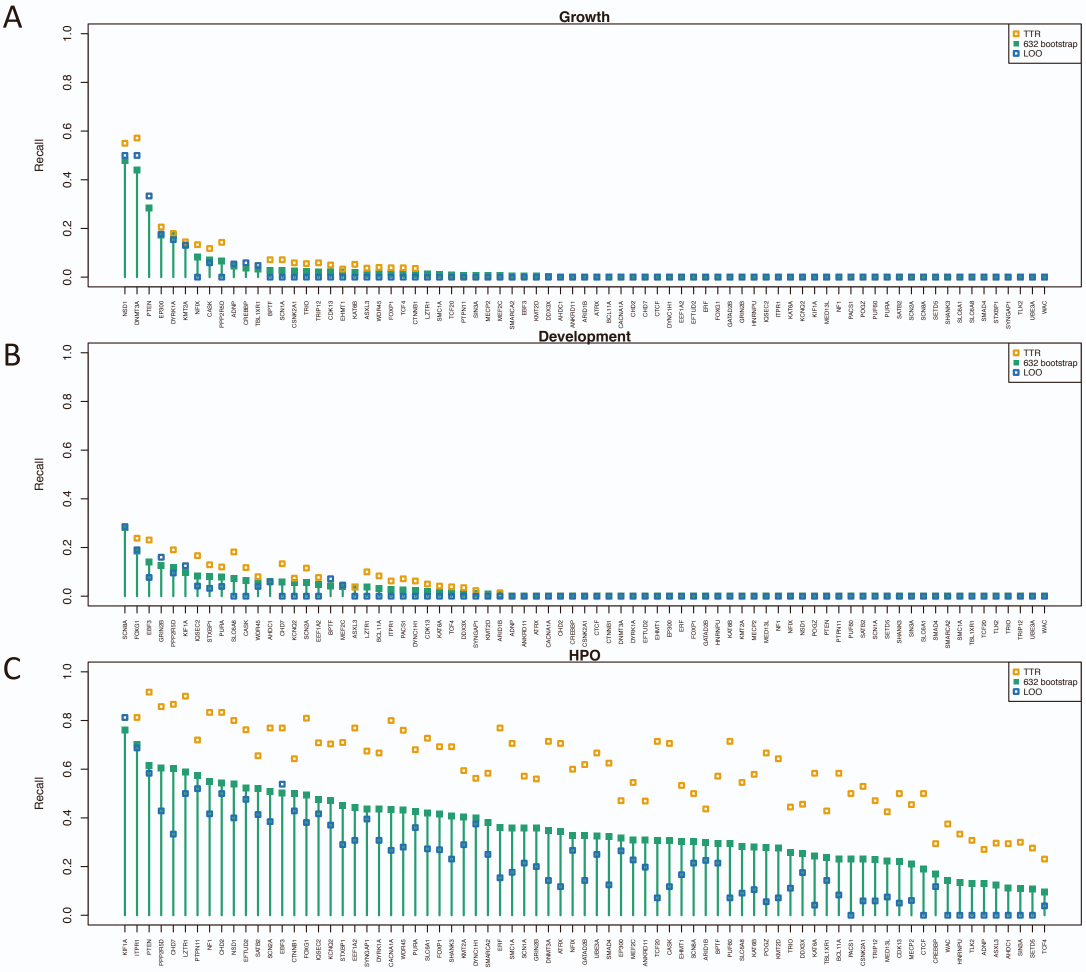

Figure S7. Bootstrap cross-validation shows a balanced estimate of recall lies between testing on training and leave-one-out rates.

A) Recall per gene using growth data alone when testing on training data, leave-one-out and by .632 bootstrap. Vertical bars and filled symbols highlight .632 recall.

B) Recall per gene using development data alone when testing on training data, leave-one-out and by .632 bootstrap. Vertical bars and filled symbols highlight .632 recall.

C) Recall per gene using HPO data alone when testing on training data, leave-one-out and by .632 bootstrap. Vertical bars and filled symbols highlight .632 recall.
